# The p38α Stress Kinase Suppresses Aneuploidy Tolerance by Inhibiting Hif-1α

**DOI:** 10.1016/j.celrep.2018.09.060

**Published:** 2018-10-16

**Authors:** Susana Simões-Sousa, Samantha Littler, Sarah L. Thompson, Paul Minshall, Helen Whalley, Bjorn Bakker, Klaudyna Belkot, Daniela Moralli, Daniel Bronder, Anthony Tighe, Diana C.J. Spierings, Nourdine Bah, Joshua Graham, Louisa Nelson, Catherine M. Green, Floris Foijer, Paul A. Townsend, Stephen S. Taylor

**Affiliations:** 1 Division of Cancer Sciences, Faculty of Biology, Medicine and Health, University of Manchester, Manchester Cancer Research Centre, Wilmslow Road, Manchester M20 4QL, UK; 2 European Research Institute for the Biology of Ageing (ERIBA), University of Groningen, University Medical Center Groningen, 9713 AV Groningen, the Netherlands; 3 Wellcome Centre Human Genetics, University of Oxford, Roosevelt Drive, Oxford OX3 7BN, UK

**Keywords:** mitosis, chromosome instability, aneuploidy

## Abstract

Deviating from the normal karyotype dramatically changes gene dosage, in turn decreasing the robustness of biological networks. Consequently, aneuploidy is poorly tolerated by normal somatic cells and acts as a barrier to transformation. Paradoxically, however, karyotype heterogeneity drives tumor evolution and the emergence of therapeutic drug resistance. To better understand how cancer cells tolerate aneuploidy, we focused on the p38 stress response kinase. We show here that p38-deficient cells upregulate glycolysis and avoid post-mitotic apoptosis, leading to the emergence of aneuploid subclones. We also show that p38 deficiency upregulates the hypoxia-inducible transcription factor Hif-1α and that inhibiting Hif-1α restores apoptosis in p38-deficent cells. Because hypoxia and aneuploidy are both barriers to tumor progression, the ability of Hif-1α to promote cell survival following chromosome missegregation raises the possibility that aneuploidy tolerance coevolves with adaptation to hypoxia.

## Highlights

• The p38 stress response kinase promotes apoptosis following aneuploidy induction
• Aneuploidy-induced metabolic collapse is ameliorated upon inhibition of p38
• p38 deficiency upregulates Hif-1α, buffering aneuploidy-induced metabolic collapse
• Aneuploidy tolerance may have coevolved with adaptation to hypoxia

## Graphical Abstract

**Figure:**
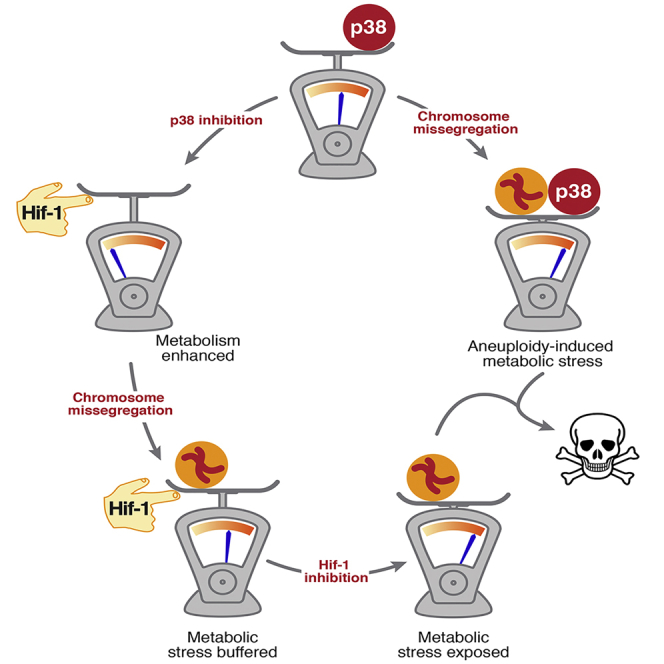

## Teaser

Simões-Sousa et al. show that chromosome missegregation induces metabolic collapse and apoptosis, mediated by the p38 stress response kinase. Inhibiting p38 elevates Hif-1α, boosts glycolysis, and limits metabolic collapse, in turn allowing expansion of aneuploid clones. Adapting to hypoxia during tumor development may therefore also permit aneuploidy tolerance.

## Introduction

Aneuploidy, a deviation from the normal karyotype, arises following chromosome missegregation during mitosis and meiosis (Holland and Cleveland, 2012, Santaguida and Amon, 2015). This leads to dramatic changes in gene dosage, unbalancing hundreds to thousands of genes, in turn leading to both chromosome-specific and global changes in transcript levels (Sheltzer et al., 2012, Torres et al., 2007, Williams et al., 2008). While post-transcriptional controls can partially buffer the effect on the proteome (Dephoure et al., 2014, Donnelly and Storchová, 2014), it is not surprising that gaining or losing an entire chromosome has a profound effect on cellular physiology (Gordon et al., 2012, Oromendia and Amon, 2014). Indeed, aneuploidy appears to decrease the robustness of many, if not all, biological processes (Beach et al., 2017).

The cellular consequences of aneuploidy include proteotoxic, lysosomal, and oxidative stress, all contributing to altered metabolism, suppressed proliferation, and reduced fitness (Oromendia et al., 2012, Pfau et al., 2016, Santaguida et al., 2015, Sheltzer et al., 2017, Stingele et al., 2012, Williams et al., 2008). In turn, this has severe organism-level consequences. In humans, aneuploidy is the leading cause of spontaneous abortions, and of the autosomal trisomies, only Down syndrome individuals (trisomy 21) are able to reach adulthood (Roper and Reeves, 2006). Aneuploidy is also associated with aging. Mice hypomorphic for the chromosome segregation regulator *Bub1b* develop aneuploidy and aging-related phenotypes including cataracts and muscle wasting (Baker et al., 2004). In humans, *BUB1B* mutation leads to mosaic variegated aneuploidy (MVA), a rare disorder characterized by progeroid features and early death (Hanks et al., 2004).

In some circumstances, aneuploidy can be advantageous. When yeast cells are placed under strong selective pressure, aneuploidy can emerge as an adaptive evolutionary response (Rancati et al., 2008). Aneuploidy can also confer a selective advantage to human cells cultured under nonstandard conditions (Rutledge et al., 2016). Moreover, genomic instability and aneuploidy are hallmarks of cancer (Hanahan and Weinberg, 2011). Experimentally inducing aneuploidy can facilitate tumor evolution in mouse models (Funk et al., 2016), and individuals with MVA are cancer prone (Hanks et al., 2004). Moreover, in non-small-cell lung cancer, elevated copy-number heterogeneity, an indicator of chromosomal instability, is associated with shorter relapse-free survival (Jamal-Hanjani et al., 2017). This paradox (that aneuploidy can inhibit fitness in some contexts but be advantageous in others) is further illustrated by the ability of some normal cell types to tolerate aneuploidy. Hepatocytes frequently become tetraploid and then undergo multipolar divisions, yielding aneuploid daughters (Duncan et al., 2010). Moreover, inactivating the spindle checkpoint gene *Mad2* in mouse skin reveals different responses to aneuploidy; while proliferating epidermal cells survive, hair follicle stem cells are eliminated via apoptosis (Foijer et al., 2013). A key question therefore is what are the context specific mechanisms that allow cells to either tolerate or be intolerant of aneuploidy?

One factor implicated in aneuploidy tolerance is the p53 tumor suppressor; for example, mutating p53 in human intestinal stem cell cultures facilitates the emergence of highly aneuploid organoids (Drost et al., 2015). In addition, p53 is activated following various mitotic abnormalities (Ditchfield et al., 2003, Lambrus et al., 2015, Lanni and Jacks, 1998). However, it is not clear whether this is a direct effect of aneuploidy or an indirect consequence of DNA damage that occurs when chromosomes become trapped in the cleavage furrow or in micronuclei (Crasta et al., 2012, Janssen et al., 2011, Li et al., 2010, Thompson and Compton, 2010). Indeed, a recent study showed that while p53 limits proliferation following errors that lead to structural rearrangements, it is not always activated by whole-chromosome aneuploidies (Soto et al., 2017).

The p38 mitogen-activated protein kinase (MAPK) has also been implicated in mitotic and post-mitotic responses (Lee et al., 2010, Takenaka et al., 1998, Vitale et al., 2008), with two separate studies showing that pharmacological inhibition of p38 overrides the p53-dependent cell-cycle block following prolonged mitosis or chromosome missegregation (Thompson and Compton, 2010, Uetake and Sluder, 2010). Chromosome instability also activates MAPK signaling in flies, in this case via JNK (Dekanty et al., 2012). Because p38 is activated by various stresses, including proteotoxic and oxidative stress (Cuadrado and Nebreda, 2010, Cuenda and Rousseau, 2007), these observations raise the possibility that p38 may also play a role in aneuploidy tolerance upstream of p53. Here, we explore this possibility further using pharmacological and CRISPR/Cas9 (clustered regularly interspaced short palindromic repeats/Cas9) approaches to suppress p38 function, followed by single-cell analysis to study mitotic cell fate.

## Results

### p38 Inhibition Suppresses Apoptosis following Chromosome Missegregation

To study aneuploidy tolerance, we focused on HCT116 cells, a near-diploid, chromosomally stable colon cancer cell line with robust post-mitotic mechanisms that limit proliferation of aneuploid daughters (Lengauer et al., 1997, Thompson and Compton, 2010). To study the role of p53, we employed *TP53*^−/−^ cells generated by adeno-associated virus (AAV)-enhanced gene targeting (Bunz et al., 2002), and to inhibit p38, we used the ATP-competitive inhibitor SB203580 (Cuenda et al., 1995). To induce aneuploidy, we used an inhibitor of the Mps1 spindle checkpoint kinase, AZ3146, to trigger chromosome missegregation (Hewitt et al., 2010). Analysis of parental cells exposed to AZ3146 showed a marked increase in cells with sub-2n DNA contents, indicating apoptosis (Figure S1A). This was ameliorated in *TP53*^−/−^ cells and by SB203580, consistent with p53 and p38 being required for apoptosis following chromosome missegregation. *TP53*^−/−^ and SB203580-exposed cells also entered additional cell cycles and accumulated aneuploidies following Mps1 inhibition (Figure S1B).

**Figure 1:**
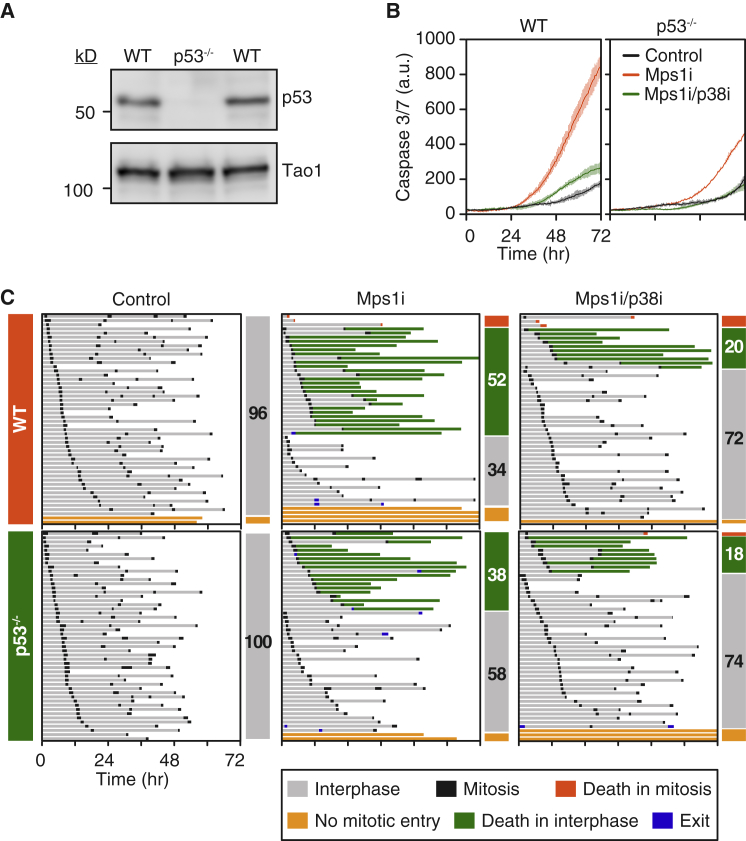
SB203580 Suppresses Apoptosis following Chromosome Missegregation (A) Immunoblots showing p53 loss following CRISPR/Cas9-mediated mutation of *TP53*. (B and C) Line graphs (B) and cell fate profiles (C) showing that p53 mutation and exposure to the p38 inhibitor SB203580 suppress apoptosis induced by the Mps1 inhibitor AZ3146. In (B), values show mean ± SD from three technical replicates and are representative of three independent experiments. In (C), numbers in bars indicate the percentage of cells exhibiting the fate indicated by bar color. See also Figure S1.

The AAV-generated HCT116 *TP53*^−/−^ cells have been extensively passaged and, due to their mismatch repair defect, have likely undergone extensive genetic drift (Lengauer et al., 1997). Indeed, introducing p53 transgenes into these cells results in lethality (D. Jackson, personal communication). Therefore, to analyze p38 and p53 in more closely matched cells, we generated *TP53*^−/−^ cells using CRISPR/Cas9-mediated gene editing and analyzed them at low passage (Figure 1A). Importantly, the CRISPR-generated *TP53*^−/−^ cells remained near diploid (Figure S1C), confirming that p53 loss is insufficient to induce aneuploidy, at least in HCT116 cells (Bunz et al., 2002).

To measure cell-cycle timing, apoptosis, and post-mitotic behavior, we used time-lapse imaging in conjunction with a fluorescent caspase-3/7 reporter (Topham et al., 2015). AZ3146 induced extensive apoptosis in parental cells, and again, this was ameliorated by p53 loss and SB203580 (Figure 1B). Cell fate profiling showed that 52% of parental cells underwent apoptosis following mitosis (Figure 1C), with 36% dying after the first mitosis and only 37% entering a second mitosis (Figure S1D). While p53 mutation had a modest effect, reducing apoptosis to 38%, SB203580 had a more substantial effect, reducing apoptosis to 20%, with 53% of cells entering a second mitosis (Figure 1C). Inactivating p53 enhanced the SB203580 effect (e.g., increasing the number of cells entering a second mitosis from 53% to 70%) (Figure S1D). Thus, we conclude that while both p53 and p38 enhance apoptosis following chromosome missegregation, analysis of more closely matched lines indicates that inhibition of p38 yields a more penetrant effect.

### p38 Is Activated following Induction of Whole-Chromosome Aneuploidy

**Figure 2:**
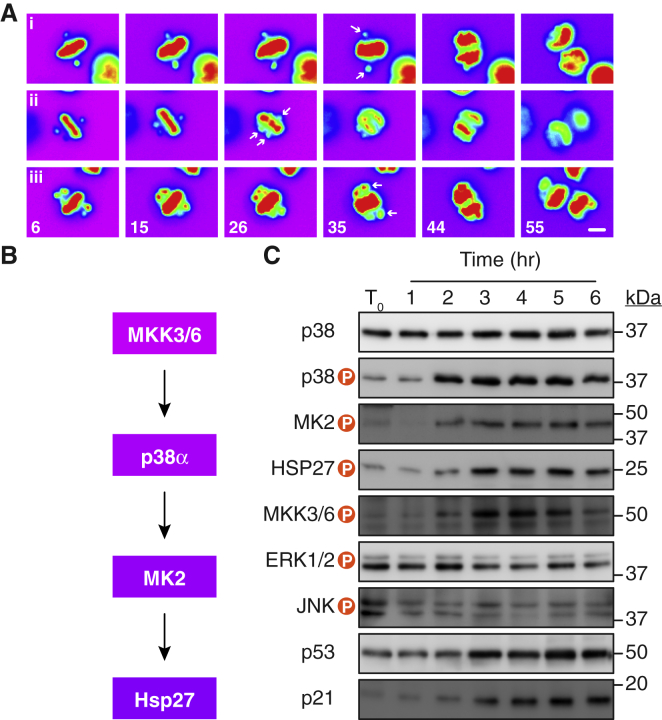
p38 Is Activated following Induction of Whole-Chromosome Aneuploidy (A) Time-lapse image sequences of HCT116 cells expressing GFP-H2B exposed to the Cenp-E inhibitor GSK923295 then AZ3146 to induce missegregation of polar chromosomes. Numbers represent minutes after imaging started; AZ3146 was added at t = 9 min. Scale bar, 10 μm. (B) Canonical p38 MAPK pathway showing upstream regulators and downstream targets. (C) Immunoblots of post-mitotic cells harvested at the time points indicated following exposure to GSK923295 then AZ3146. See also Figure S2.

Because SB203580 suppresses apoptosis following spindle assembly checkpoint (SAC) override, we asked if the canonical p38 pathway was activated following chromosome missegregation. However, SAC override induces a variety of mitotic abnormalities, some of which can lead to DNA damage (Crasta et al., 2012, Janssen et al., 2011). Indeed, inhibition of DNA-PK, which is required for non-homologous end joining (NHEJ), further suppressed apoptosis in AZ3146-treated cells exposed to SB203580 (Figure S1A). By contrast, ATM and ATR inhibitors had little effect (data not shown). Interestingly, NHEJ is required for chromosome repair following chromothripsis, localized genomic rearrangements that follow incorporation of missegregated chromosomes into micronuclei (Ly et al., 2017). Therefore, to minimize DNA damage, we employed GSK923295, which inhibits the Cenp-E kinesin, allowing most chromosomes to align at the cell equator but blocking a small number near the spindle poles (Wood et al., 2010). Upon triggering anaphase via Mps1 inhibition, these polar chromosomes are missegregated without being trapped in the spindle midzone, which could otherwise lead to DNA damage, chromosome breakage, and structural aneuploidy (Bennett et al., 2015, Soto et al., 2017). To evaluate this approach in HCT116, cells harboring a GFP-tagged histone were exposed to GSK923295 and analyzed by time-lapse microscopy. Mitotic cells with polar chromosomes were readily apparent, and addition of AZ3146 triggered their missegregation without chromosome trapping (Figure 2A).

Having confirmed that sequential inhibition of Cenp-E and Mps1 induces aneuploidy in HCT116 cells, mitotic cells exposed to GSK923295 were isolated by selective detachment, re-plated in AZ3146, harvested at various time points, and then analyzed by immunoblotting to interrogate the p38 pathway. Note that p38 is phosphorylated by upstream kinases MKK3/6, in turn leading to phosphorylation of MK2 and Hsp27 (Figures 2B and S2A). 2 hr after driving GSK923295-arrested cells into anaphase, levels of phospho-p38 notably increased, as did phosphorylated MKK3/6, MK2, and Hsp27 (Figure 2C). p53 and p21 also increased over the 6-hr time course, although this increase was slightly delayed compared to p38 activation. However, the parallel ERK and JNK pathways did not show signs of activation. Importantly, under these conditions, DNA damage was not apparent, and inhibitors targeting ATM, ATR, and DNA-PK did not suppress p38 activation (Figures S2B and S2C). While these observations support the notion that p38 is activated upon aneuploidy induction, a caveat arises, because a mitotic delay can be sufficient to induce a p38-dependent response (Uetake and Sluder, 2010). To address this, we released cells from a mitotic block with and without the Mps1 inhibitor. Importantly, only in the presence of the AZ3146 did we observe elevated p38 phosphorylation (Figures S2C and S2D). Moreover, driving cells from the mitotic block with an Aurora B inhibitor, thereby inducing tetraploidy rather than aneuploidy, failed to activate p38 (Figure S2C).

### p38α Promotes Apoptosis following Chromosome Missegregation

**Figure 3:**
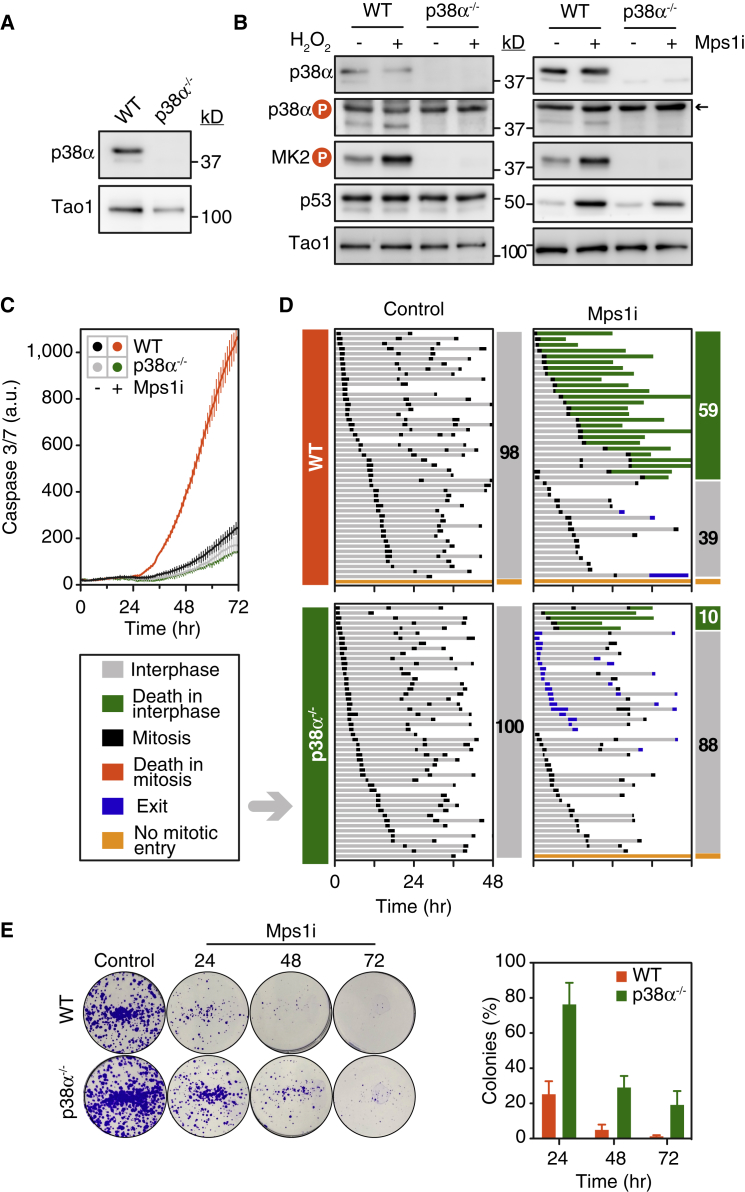
p38α Promotes Apoptosis following Chromosome Missegregation (A) Immunoblot showing p38α loss following CRISPR/Cas9-mediated mutation of *MAPK14*. (B) Immunoblots of parental and p38α null cells exposed to H_2_O_2_ for 30 min (left) or AZ3146 for 24 hr (right). Arrow highlights a background band. (C–E) Line graphs (C), cell fate profiles (D), and colony formation assay (E) showing suppression of AZ3146-induced apoptosis in p38α null cells. In (C), values show mean ± SD from three technical replicates and are representative of three independent experiments. Quantitation in (E) shows the mean ± SD derived from two independent experiments. See also Figure S3.

To validate our observations derived from pharmacological inhibition of p38, we used CRISPR/Cas9 to mutate *MAPK14*, which encodes p38α, the isoform expressed in most cell types (Cuenda and Rousseau, 2007). Using two different single guide RNAs (sgRNAs) we generated two independent clones devoid of p38α (Figures 3A and S3A). These lines lacked p38 signaling, as evidenced by lack of MK2 phosphorylation following exposure to hydrogen peroxide (Figure 3B). Time-lapse imaging showed that AZ3146-induced apoptosis was suppressed to near basal levels in both p38α null clones (Figure 3C and S3B). Cell fate profiling confirmed this. Within 48 hr, 59% of parental cells underwent post-mitotic death; by contrast, only 10% of the p38α null cells died (Figure 3D). Also, whereas only 18% of parental cells entered a second mitosis, 50% of the p38α null cells did so (Figure S3C). The independent p38α null clone was also resistant; only 4% of cells died, while 82% entered a second mitosis (Figures S3B and S3C). Other cell-cycle parameters, including mitotic duration and time between successive mitoses, appeared unaffected by p38α mutation (Figure S3D). To test whether apoptosis avoidance led to longer-term survival, cells exposed to AZ3146 for 24, 48, and 72 hr were allowed to grow out into colonies. Notably, the p38α null clone yielded more colonies across the entire time course (Figure 3E). Thus, we conclude that p38α promotes post-mitotic apoptosis following chromosome missegregation. Note that some p38α null cells failed cell division in the presence of AZ3146 due to abscission failure, yielding tetraploid cells; we discuss this issue below. To ascribe the p38α-dependent effect to an aneuploidy response, as opposed to DNA damage, we exposed p38α null to GSK923295 and AZ3146 as described above (Figure 2). However, to avoid complications associated with a mitotic delay, we exposed the cells to both inhibitors simultaneously (Soto et al., 2017). Time-lapse imaging showed that 63% of cells entered anaphase with unaligned chromosomes and that 88% exited mitosis within 90 min (Figure S3E). (Note that previously, post-mitotic responses only manifested when mitosis was delayed beyond 90 min; Uetake and Sluder, 2010.) Under these conditions, p38α loss also suppressed apoptosis and enhanced colony formation (Figures S3F–S3H), consistent with aneuploidy being a key driver of post-mitotic stress.

### p38α Promotes p53 Stabilization following Chromosome Missegregation

**Figure 4:**
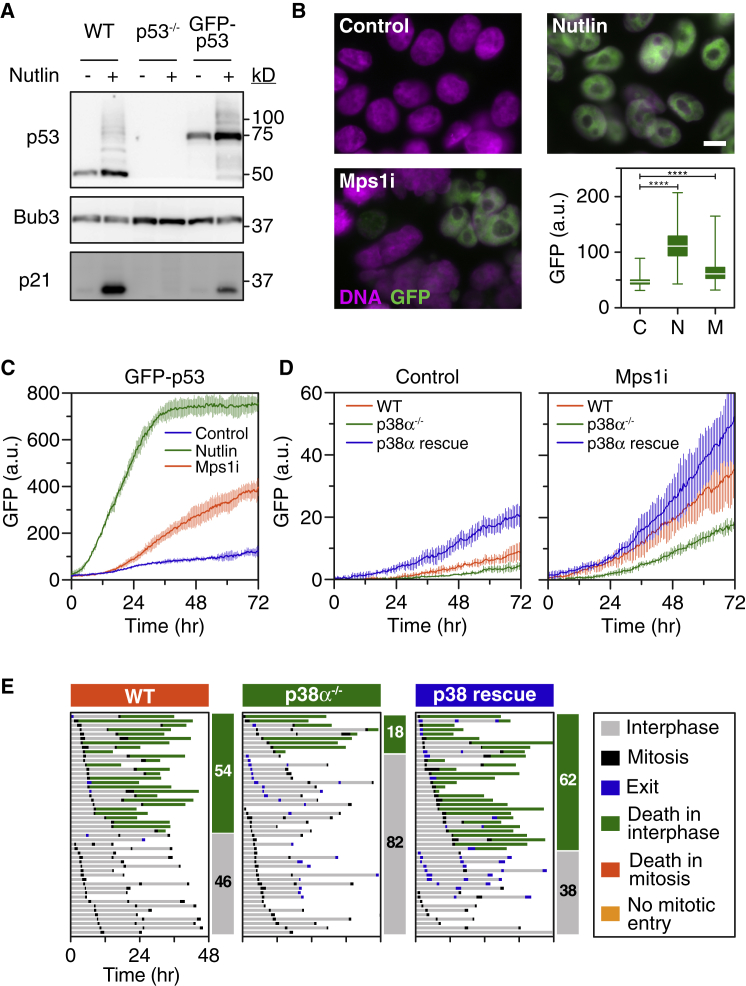
p38α Promotes p53 Stabilization following Chromosome Missegregation (A) Immunoblot showing expression of a GFP-p53 fusion protein following CRISPR/Cas9-mediated targeting of *TP53*. (B) Immunofluorescence images and quantitation showing nuclear GFP-p53 following exposure to Nutlin-3 and AZ3146. Scale bar, 10 μm. Box and whisker plot shows median, interquartile range, and full range from 431 cells per condition from one biological replicate. ^∗∗∗∗^p < 0.0001. (C) Line graph showing accumulation of green fluorescence in GFP-p53 cells following exposure to Nutlin-3 and AZ3146. (D) Line graphs showing reduced accumulation of green fluorescence in p38α null cells exposed to AZ3146 and restoration following p38α rescue. In (C) and (D) values show mean ± SD from three technical replicates and is representative of three independent experiments. (E) Cell fate profiles of GFP-p53 cells showing suppression of AZ3146-induced apoptosis in p38α null cells and restoration in p38α rescue cells. See also Figure S4.

As alluded above, p53 accumulation was attenuated in p38α null cells (Figure 3B). To measure p53 accumulation more directly, we integrated GFP into the endogenous *TP53* using CRISPR/Cas9. Immunoblotting confirmed that all the detectable p53 was expressed as a GFP fusion, suggesting that both *TP53* alleles had been modified (Figure 4A). Importantly, like untagged p53, the GFP fusion also accumulated upon Nutlin-3-mediated inhibition of Mdm2. Moreover, fluorescence microscopy and time-lapse imaging demonstrated nuclear accumulation of GFP in response to both Nutlin-3 and AZ3146 (Figures 4B and 4C). To determine functionality of the GFP-p53 fusion, we analyzed proliferation in the presence and absence of Nutlin-3. As expected, Nutlin-3 inhibited proliferation of parental cells, but not p53 null cells (Figure S4A). Importantly, Nutlin-3 suppressed proliferation of GFP-p53 cells, demonstrating functionality of the fusion. Nutlin-3 also induced p21 in GFP-p53 cells, further supporting this conclusion (Figure 4A). Having validated the GFP-p53 biosensor, we mutated *MAPK14* with CRISPR/Cas9 (Figure S4B) and used time-lapse imaging to measure GFP fluorescence upon exposure to AZ3146. Over 72 hr, GFP increased in p38α-proficient cells but was attenuated in p38α null cells (Figure 4D). To confirm this reflected the loss of p38α, we restored its function by stably transfecting a p38α cDNA into GFP-p53 *MAPK14*^−/−^ cells (Figure S4B). Importantly, this restored accumulation of GFP-p53 (Figure 4D). Thus, we conclude that p38α does indeed contribute to p53 accumulation following chromosome missegregation. Whether this reflects a direct effect is unclear. Note also that the p38α cDNA restored post-mitotic apoptosis in AZ3146-treated cells; whereas only 18% of the p38α null cells died, 62% did so in the p38α rescue line (Figure 4E), confirming that reduced post-mitotic apoptosis in *MAPK14*^−/−^ cells is indeed due to loss of p38α function.

### p38α Promotes Post-mitotic Apoptosis by Suppressing Hif-1α

**Figure 5:**
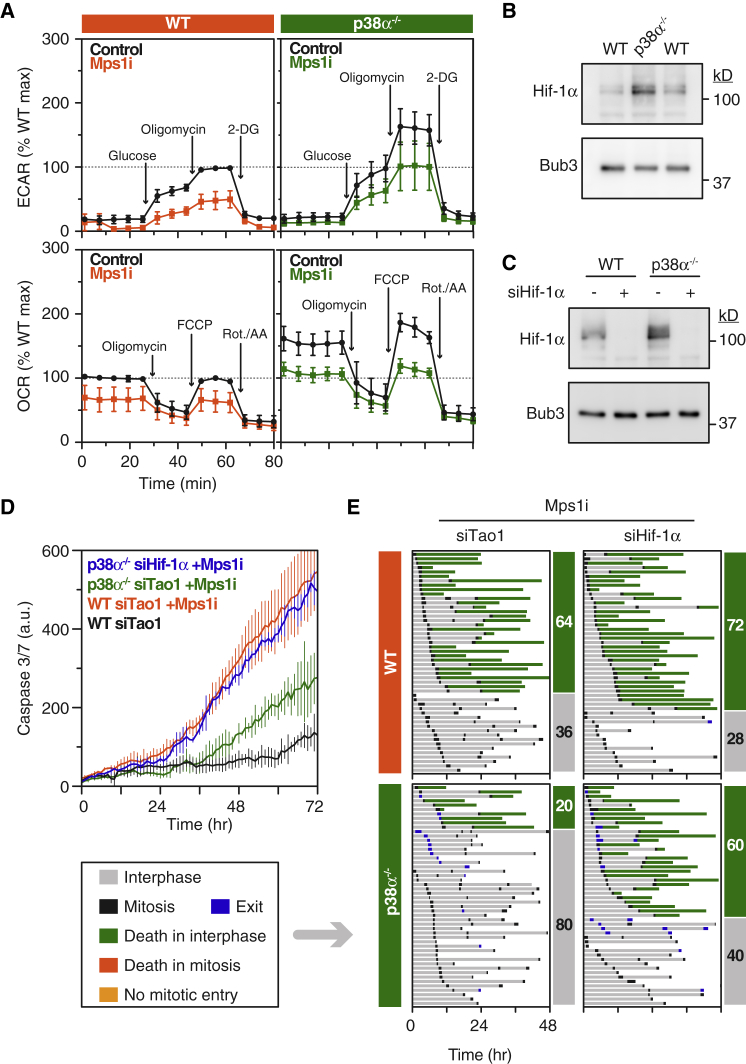
p38α Promotes Post-mitotic Apoptosis by Suppressing Hif-1α (A) Line graphs showing the extracellular acidification rate (ECAR) and oxygen consumption rate (OCR) in parental and p38α null cells after exposure to AZ3146 for 24 hr. Values show mean ± SEM from three independent experiments (see Table S1), normalized to the maximal value observed in untreated parental cells. (B) Immunoblot showing elevated Hif-1α in p38α null cells. (C) Immunoblot showing RNAi-mediated repression of Hif-1α; note also elevated Hif-1α in control p38α null cells. (D and E) Line graphs (D) and fate profiles (E) showing restoration of AZ3146-mediated apoptosis in p38α null cells following siHif-1α. In (D), values show mean ± SD from two technical replicates and is representative of three independent experiments. See also Figure S5.

To dissect how p38α promotes post-mitotic apoptosis, we considered several approaches. Because proteomic analyses of aneuploid cells highlight alterations in oxidative stress and metabolism (Dephoure et al., 2014, Donnelly and Storchová, 2014), we turned to real-time measurement of metabolic parameters using Seahorse XF technology. Parental and p38α null cells were treated with AZ3146 for 24 hr, and then the extracellular acidification rate (ECAR) and oxygen consumption rate (OCR) were analyzed. Notably, exposing parental cells to AZ3146 suppressed both ECAR and OCR, indicating suppressed glycolysis and mitochondrial respiration, respectively (Figure 5A; Table S1). More strikingly, both parameters were elevated in p38α null cells, and while exposure to AZ3146 still suppressed both ECAR and OCR, they were maintained at values observed in untreated controls. Based on this, we speculated that by enhancing metabolic parameters, p38α mutation allows cells to buffer the consequences of chromosome missegregation, thus enhancing survival.

To test this notion, we turned to Hif-1, a heterodimeric transcription factor and master regulator of glycolysis (Porporato et al., 2011). Notably, Hif-1α was elevated in both p38α null clones (Figures 5B and S5A). Moreover, it was elevated in GFP-p53 *MAPK14*^−/−^ cells, and restoring p38α suppressed Hif-1α to basal levels (Figure S4B). To test whether elevated Hif-1α caused apoptosis suppression in p38α nulls, we repressed Hif-1α using RNAi (Figure 5C). Hif-1α depleted cells were then analyzed by time-lapse microscopy, with Tao1 small interfering RNAs (siRNAs) serving as a negative control. Consistent with observations above, the extensive apoptosis induced by AZ3146 in controls was suppressed by p38α mutation (Figure 5D). Importantly, Hif-1α RNAi restored apoptosis in p38α null cells. This was confirmed by cell fate profiling; while Hif-1α RNAi had only a marginal effect on parental cells, increasing post-mitotic apoptosis from 64% to 72%, it had a dramatic effect on p38α null cells, increasing apoptosis from 20% to 60% (Figure 5E). Analysis of the second *MAPK14*^−/−^ clone yielded a similar result, with Hif-1α RNAi restoring AZ3146-induced apoptosis from 26% to 54% (Figure S5B). Thus, we conclude that suppressed post-mitotic apoptosis in p38α null cells can be explained by elevated Hif-1α. Based on the large body of evidence demonstrating that Hif-1α drives glycolytic pathways (Kim and Dang, 2006, Porporato et al., 2011, Semenza, 2011), one possible explanation for these observations is that by enhancing glycolysis, elevated Hif-1α allows cells to buffer the metabolic consequences that arise following chromosome missegregation.

### p38α-Deficient Cells Accumulate Whole-Chromosome Aneuploidies

Our observations demonstrate that p38α promotes apoptosis following chromosome missegregation (Figures 3, 4, and 5) and that p38α’s role enhances longer-term survival following spindle checkpoint override (Figure 3E). To determine whether the p38α-deficient survivors are indeed aneuploid and that they retained deviant karyotypes following clonal expansion, parental and p38α null cells were exposed to AZ3146 for 24 hr, expanded for a further 25 days, and then analyzed independently using two orthogonal approaches, namely traditional chromosome spreads (Tighe et al., 2004) and single-cell whole-genome sequencing (scWGS) (Bakker et al., 2016, van den Bos et al., 2016). As expected, untreated parental cells had near-diploid chromosome counts, with 67% possessing the modal chromosome number (Figure S6A). By contrast, chromosome numbers in AZ3146-treated cultures deviated considerably, ranging from 40 to 92. Interestingly, while untreated p38α null cells were largely near diploid, only 19% had the modal chromosome count, suggesting that p38α null cells accumulate aneuploidies without experimentally inducing chromosome missegregation.

**Figure 6:**
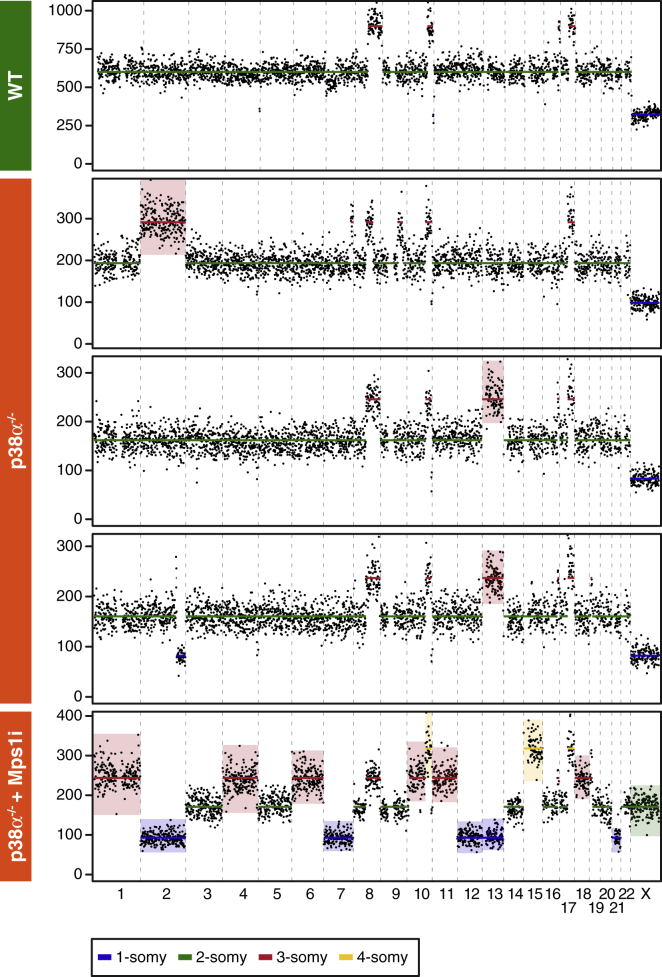
p38α-Deficient Cells Accumulate Whole-Chromosome Αneuploidies Genome-wide chromosome copy-number profile of parental and p38α null cells as determined by single-cell sequencing, with colored boxes highlighting whole-chromosome aneuploidies not observed in parental cells. See also Figure S6.

To analyze the populations by scWGS, G1 cells were isolated by flow sorting and then subjected to next-generation sequencing. In untreated parental cells, we observed clonal copy-number gains affecting chromosomes 8, 10, 16, and 17 (Figure S6B). Note that these gains reflect translocations as previously reported for HCT116 (Abdel-Rahman et al., 2001) and were observed in our multiplex fluorescence *in situ* hybridization (M-FISH) analysis (see below). Beyond this baseline, out of 23 untreated p38α null cells, three had a trisomy affecting either chromosome 2 or 13; and out of 22 AZ3146-treated cells, one had a highly deviant karyotype trisomic for six chromosomes and monosomic for another five (Figure 6). During the flow sorting, we observed a substantial number of AZ3146-treated p38α null cells with 4N DNA contents (not shown), consistent with abscission failure highlighted by the cell fate profiling (Figure 3D). scWGS confirmed that these cells were near tetraploid but with a number of chromosome losses (Figure S6B). Nevertheless, scWGS analysis of the near-diploid cells supports the notion that p38α null cells are more likely to accumulate aneuploidies.

### Pharmacological Inhibition of p38 Facilitates Expansion of Aneuploid Clones

**Figure 7:**
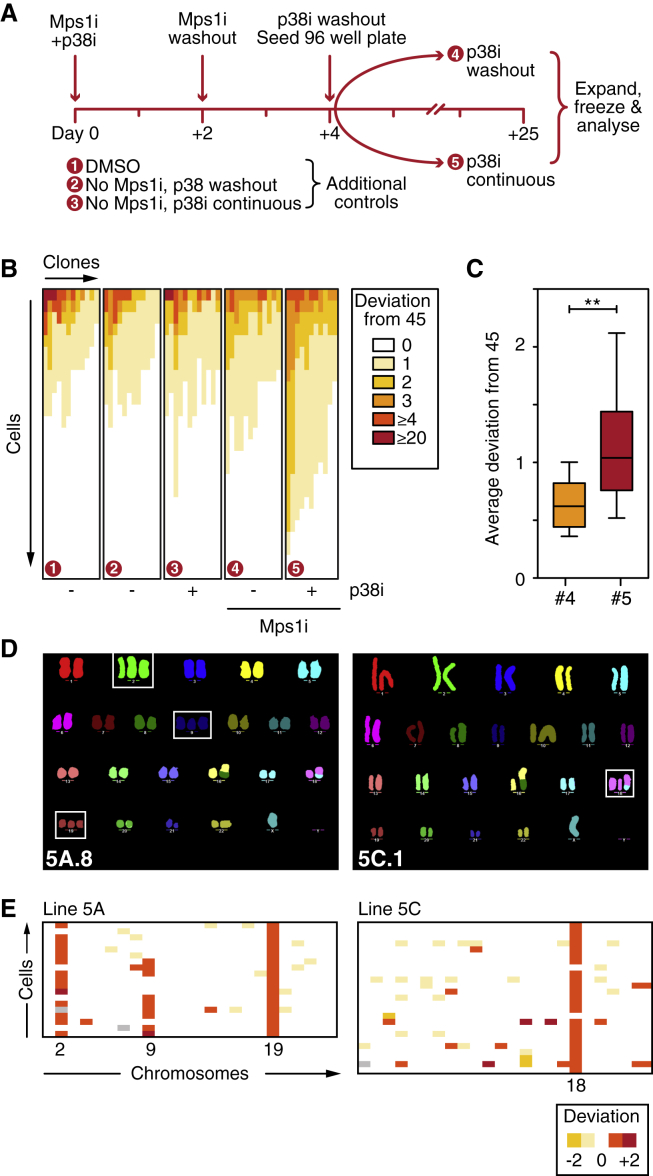
Pharmacological Inhibition of p38 Facilitates Expansion of Aneuploid Clones (A) Experimental design generating clones in the presence or absence of the p38 inhibitor SB203580 following exposure to AZ3146. (B) Chromosome counts showing the deviation from the modal number of 45, analyzing 25 spreads from at least 10 independent clones for each condition. (C) Box and whisker plot showing the average deviation from 45 for clones exposed to AZ3146 ± SB203580. ^∗∗^p < 0.01. (D) Representative M-FISH karyotypes from clones 5A and 5C, both generated in the continuous presence of SB203580. Boxes highlight whole-chromosome aneuploidies. (E) Quantitation of M-FISH karyotypes showing recurrent trisomies for chromosome 2, 9, and 19 (clone 5A) and chromosome 18 (clone 5C). See also Figure S7.

While the scWGS identified aneuploidies in the p38α null population, we noted three limitations with this experiment. First, untreated p38α nulls already showed signs of aneuploidy. Second, if aneuploidy has a fitness cost, it may be underrepresented when analyzing populations due to outcompeting diploid survivors. Finally, by inducing tetraploidy, p38α mutation might provide AZ3146-treated cells with an alternative survival mechanism not related to aneuploidy tolerance per se. To address these issues, we returned to pharmacological inhibition of p38 in parental cells. Note that in contrast to p38α mutation, SB203580 does not induce cell division failure in Mps1-inhibited cells (Figure 1). Cells were treated with AZ3146 for 48 hr and allowed to recover for a further 48 hr, and then single cells were expanded in the presence or absence of SB203580 before independent analysis using two orthogonal approaches: chromosome counting and M-FISH (Figure 7A). Chromosome counts showed that substantially more SB203580-treated cells deviated from the mode of 45 (Figure 7B). Indeed, the average deviation in controls was 0.63 compared to 1.11 in the SB203580-treated arm (Figure 7C). M-FISH confirmed that SB203580-treated clones were indeed aneuploid, with clone 5A trisomic for chromosomes 2, 9, and 19 and clone 5C trisomic for chromosome 18 (Figure 7D). By contrast, control clones exhibited the typical HCT116 karyotype (Figure S7). Interestingly, while all cells in 5A were trisomic for chromosome 19, chromosomes 2 and 9 were more heterogeneous, indicative of chromosome instability (Figure 7E). Nevertheless, we conclude that pharmacological inhibition of p38 following chromosome missegregation facilitates the emergence of aneuploid clones.

## Discussion

As cancer cells acquire the characteristics that distinguish them from normal cells, aneuploidy emerges as a common feature (Sansregret et al., 2018). However, some cancers retain near-diploid karyotypes because they retain segregation fidelity and/or because they do not tolerate aneuploid genomes. This is illustrated by colorectal cancers, which broadly fall into two classes: those that exhibit microsatellite instability (MIN; 20%) and remain near diploid and those that are microsatellite stable but display chromosome instability (CIN; 80%) and acquire highly divergent karyotypes (Lengauer et al., 1997, López-García et al., 2017). Due to this dichotomy, MIN cells are tractable model systems for studying both segregation fidelity and aneuploidy tolerance, and indeed, several pathways that contribute to CIN have now been identified (Ertych et al., 2014, Stolz et al., 2010). More recently, the ability of MIN cells to mount robust post-mitotic responses following experimental induction of chromosome missegregation has been exploited to shed light on aneuploidy tolerance. For example, inhibiting *BCL9L* in HCT116 cells suppresses apoptosis following spindle checkpoint override, permitting survival of aneuploid cells (López-García et al., 2017). Interestingly, this is only partially explained by an effect on p53; *BCL9L* mutation prevents caspase-2-mediated cleavage of BID, thus suppressing apoptosis regardless of p53 status. Similarly, a genome-wide screen for Taxol sensitizers in another MIN line, RKO, identified a MYC-dependent, p53-independent apoptosis module that eliminates cells following an aberrant mitosis (Topham et al., 2015). Here, we identify an additional mechanism, one dependent on the p38 stress response kinase, that when suppressed allows HCT116 cells to avoid apoptosis following chromosome missegregation, in turn leading to the emergence of aneuploid clones. While p38 can modulate apoptosis pathways directly (Cuadrado and Nebreda, 2010), one possible explanation for our observations is that in this context, p38 suppresses aneuploidy tolerance by suppressing Hif-1-dependent glycolytic networks. An important next step will be to explore these concepts in additional cell lines and preclinical models.

In addition to chromosome instability, another hallmark of cancer cells is altered metabolism, in particular shifting ATP generation from oxidative phosphorylation to increased glycolysis, thus taking up glucose and secreting lactate even when oxygen is present, a phenomenon known as the Warburg effect (Liberti and Locasale, 2016, Vander Heiden and DeBerardinis, 2017). Although less efficient, this shift offers cancer cells several advantages, including enhanced proliferation and biosynthesis, the ability to buffer reactive oxygen species (ROS), and, by allowing ATP production in the absence of oxygen, adaptation to hypoxia (Cairns et al., 2011). Indeed, because tumor growth leads to hypoxic microenvironments, this metabolic shift facilitates cancer cell survival in advance of neovascularization. A key driver of the shift to glycolysis is Hif-1α, which, in response to hypoxia, amplifies expression of genes encoding glucose transporters and glycolytic enzymes. Because enhanced glucose uptake and increased lactate production are also characteristics of non-transformed aneuploid cells, Amon and colleagues have explicitly noted the similarity between the Warburg effect and the metabolic changes caused by aneuploidy (Siegel and Amon, 2012, Williams et al., 2008). Moreover, analysis of copy-number variations across 15 tumor types revealed that glycolysis-associated genes are frequently amplified in tumors with high genomic instability, indicating that metabolic stress drives the evolution of highly aberrant genomes (Graham et al., 2017). Our observations also suggest a reciprocal relationship whereby not only does aneuploidy induce metabolic changes but also the Warburg effect perhaps facilitates aneuploidy tolerance. We show that glycolysis is suppressed immediately following chromosome missegregation but that when p38α is mutated, Hif-1α increases, glycolysis is enhanced, and post-mitotic apoptosis is suppressed. A causal link between elevated Hif-1α and post-mitotic survival is evidenced by the restoration of apoptosis upon RNAi-mediated repression of Hif-1α. One possibility therefore is that p38α suppresses aneuploidy tolerance by limiting the Warburg effect. Testing this notion will require analyzing other metabolic parameters, including glucose uptake, lactate production, and glycolytic flux, in the context of p38α signaling and chromosome missegregation.

p38 is activated in response to various cellular stresses, in turn modulating multiple downstream pathways, the net effect of which is highly context dependent, explaining p38∋σ role in various pathologies, including inflammation, cancer immune responses, heart disease, and neurodegeneration (Cuenda and Rousseau, 2007). We cannot therefore rule out the possibility that p38α promotes post-mitotic apoptosis via canonical stress response pathways, for example directly via p53 or the apoptosis machinery (Bulavin et al., 1999). Note however, that while p53 stabilization is attenuated in p38α mutant cells, this may also be an indirect effect of elevated Hif-1α. Also, because p38 inhibition suppresses post-mitotic apoptosis more potently than p53 mutation, the effect of p38 is unlikely to be exclusively via p53. Moreover, the notion that p38 suppresses Hif-1 is not unprecedented; it was recently shown that in *C. elegans*, under normoxic conditions, the p38 MAPK ortholog PMK-1 activates the EGL-9 prolyl hydroxylase, which triggers Hif-1 turnover (Park and Rongo, 2016). Thus, loss-of-function mutations in PMK-1, or the upstream mitogen-activated protein kinase kinase (MAPKK) ortholog SEK-1, mimic the effects of hypoxia, including nuclear accumulation of Hif-1 and upregulation of target genes. Thus, the de-repression of Hif-1α we observe following mutation of p38α in HCT116 cancer cells appears to reflect an evolutionarily conserved mechanism relevant to normal physiology. An important next step will be delineating how p38 modulates Hif-1 function in human cells.

Our cell fate profiling revealed an unexpected finding: a substantial number of p38α null cells generated by CRISPR/Cas9-mediated gene editing underwent abscission failure when exposed to the Mps1 inhibitor. While p38 has been implicated in chromosome segregation and cytokinesis (Tormos et al., 2017), the abscission failure appears to be a synthetic effect, as it was not observed in untreated p38α mutants or AZ3146-treated control cells. Importantly, the abscission failure was not reverted by expression of a p38α cDNA, suggesting that it may be the result of an off-target CRISPR/Cas9 phenomenon. This seems unlikely, as it was also observed in a second p38α null clone, albeit to a lesser extent, despite this mutation being generated using an independent sgRNA targeting *MAPK14*. One possibility that we are currently exploring is that this reflects an adaptive response to loss of p38α function during the clonal expansion following the CRISPR/Cas9 process. Another possibility is that aneuploidy induced by loss of p38 may predispose cells to cytokinesis failure, a phenomenon described in trisomic DLD-1 cells (Nicholson et al., 2015). Nevertheless, it raises the possibility that post-mitotic survival of p38α null cells is a consequence of avoiding aneuploidy by becoming tetraploid. Indeed, by buffering the damaging effects of chromosome missegregation, tetraploidy can produce viable albeit highly abnormal progeny (Holland and Cleveland, 2012, Storchova and Kuffer, 2008). Consistent with this notion, an siRNA library screen for genes that enhanced HCT116 cell survival upon exposure to AZ3146 yielded Aurora B, a cytokinesis regulator, as the top hit (data not shown). Note also that genome doubling is a frequent evolutionary stepping stone during tumorigenesis (Dewhurst et al., 2014, Galipeau et al., 1996). However, several reasons suggest that tetraploidy is insufficient to account for the enhanced survival of p38α null cells. First, as alluded above, when we analyzed an independent p38α null clone, only 12% of cells failed abscission after the first mitosis, yet 96% of the cells survived. Second, when we restored p38α or repressed Hif1α, cells that failed abscission now died. Third, upon pharmacological inhibition of p38, we did not observe abscission failure, yet cells survived and aneuploid clones emerged. Finally, p38α null cells acquired aneuploidies even when not being exposed to the Mps1 inhibitor.

To conclude, our observations have a provocative corollary relating to tumor evolution and the aneuploidy paradox. Hypoxia and the fitness cost incurred by aneuploid cells are barriers to tumor progression. However, the ability of elevated Hif-1α to permit cell survival following chromosome missegregation raises the possibility that adapting to hypoxic conditions during the early avascular phase of tumor development may also permit aneuploidy tolerance. An important next step will be to test these concepts in models that recapitulate the tumor microenvironment.

## STAR★Methods

### Key Resources Table

**Table:** 

REAGENT or RESOURCE	SOURCE	IDENTIFIER
**Antibodies**		

Goat anti-mouse IgG (HL) HRP	Invitrogen	Cat# G21040; RRID: AB_2536527
Goat anti-rabbit IgG (HL) HRP	Invitrogen	Cat# G21234; RRID: AB_2536530
Mouse monoclonal anti-ATM (phospho S1981) [10H11.E12]	Abcam	Cat# ab36810; RRID: AB_725573
Mouse monoclonal anti-HIF-1α (Clone 54)	BD Transduction Laboratories	Cat# 610958; RRID: AB_398271
Mouse monoclonal anti-p21 (F-5)	Santa Cruz Biotechnology	Cat# sc-6246; RRID: AB_628073
Mouse monoclonal anti-p38α (5F11)	Cell Signaling Technology	Cat# 9217S; RRID: AB_10691677
Mouse monoclonal anti-p53 (DO-1)	Santa Cruz Biotechnology	Cat# sc-126; RRID: AB_628082
Mouse monoclonal anti-phospho-p38α (T180/Y182)	R&D Systems	Cat# MAB8691; RRID: AB_10890618
Mouse monoclonal Chk1(G-4)	Santa Cruz technology	Cat# sc-8408; RRID: AB_627257
Phospho-Chk1 (Ser345) (133D3)	Cell Signaling Technology	Cat# 2348; RRID: AB_331212
Phospho-gamma H2AX (Ser139)	Novus Bio	Cat# NB100-384; RRID: AB_350295
Phospho-HSP27 (Ser82)	Cell Signaling Technology	Cat# 2401; RRID: AB_331644
Phospho-KAP-1 (S824)	Bethyl Laboratories	Cat# A300-767A; RRID: AB_669740
Phospho-MKK3/6 (Ser189/207)	Cell Signaling Technology	Cat# 9236; RRID: AB_491009
Phospho-p44/42 MAPK (ERK1/2)	Cell Signaling Technology	Cat# 9102; RRID: AB_330744
Phospho-SAPK/JNK (T183/Y185)	Cell Signaling Technology	Cat# 4668; RRID: AB_2307320
Rabbit anti-sheep IgG (HL) HRP	Invitrogen	Cat# 618620; RRID: AB_2533942
Rabbit monoclonal anti-ATM [Y170]	Abcam	Cat# ab32420; RRID: AB_725574
Rabbit monoclonal anti-phospho-MAPKAP-K2 (Thr334) (27B7)	Cell Signaling Technology	Cat# 3007L; RRID: AB_490936
Rabbit polyclonal anti-phospho-p53 (Ser 46)	Cell Signaling Technology	Cat# 2521P; RRID: AB_10828689
Rabbit polyclonal DNA PKcs	Abcam	Cat# ab70250; RRID: AB_1209452
Rabbit polyclonal DNA PKcs (phospho S2056) – ChIP Grade)	Abcam	Cat# ab18192; RRID: AB_869495
Rabbit polyclonal p38 MAPK	Cell Signaling Technology	Cat# 9212; RRID: AB_330713
Sheep polyclonal anti-BUB3	A. Holland and S.S.-T., unpublished data	N/A
Sheep polyclonal anti-Tao1	(Westhorpe et al., 2010)	N/A

**Bacterial and Virus Strains**		

XL1-Blue Competent Cells	Agilent Technologies	Cat# 200249

**Biological**		

N/A		

**Chemicals, Peptides, and Recombinant Proteins**		

AZ3146 (MPS1i)	Tocris Bioscience	Cat# 3994
Crystal Violet	Sigma Aldrich	Cat# C0775
DNA-PK Inhibitor II	Calbiochem	Cat# 260961
D-(+)-Glucose	Sigma Aldrich	Cat# G8644
L-Glutamine	Sigma Aldrich	Cat# 25030024
GSK923295 (CENP-Ei)	(Bennett et al., 2015)	N/A
Hoechst 33258	Sigma Aldrich	Cat# B1155
Hydrogen peroxide	Sigma Aldrich	Cat# H1009
Hygromycin B	Sigma Aldrich	Cat# 10843555001
Nocodazole	Sigma Aldrich	Cat# M1404
Nutlin-3	Sigma Aldrich	Cat# N6287
Penicillin-Streptomycin	Sigma Aldrich	Cat# 15140122
Propidium Iodide	Sigma Aldrich	Cat# P4170
RNase A	Thermo Scientific	Cat# EN0531
SB203580 (p38i)	Tocris Bioscience	Cat# 1202
Sodium Pyruvate	Sigma Aldrich	Cat# S8636
Tetracycline hydrochloride	Sigma Aldrich	Cat# T7660

**Critical Commercial Assays**		

FISH Probes 24XCyte, Human mFISH Probe Kit	Zeiss MetaSystems	Cat# D-0125-060-DI
Genomic DNA Extraction Kit	Invitrogen	Cat# 1851095
QIAprep® Spin Miniprep Kit	QIAGEN	Cat# 27106
REDTaq^®^ DNA Polymerase	Sigma Aldrich	Cat# D4309
RNeasy® Plus Mini Kit	QIAGEN	Cat# 74134
Seahorse XF Cell Mito Stress Test Kit	Agilent Technologies	Cat# 103015-100
Seahorse XF Glycolysis Stress Test Kit	Agilent Technologies	Cat# 103020-100

**Deposited Data**		

Single cell sequencing reads	European Nucleotide Archive (ENA)	Accession# PRJEB27319

**Experimental Models: Cell Lines**		

Human: HCT116 (male)	American Type Culture Collection	Cat# HCT-116; RRID: CVCL_0291
Human: HCT116 *TP53*^*−/−*^ (male)	A gift from Bert Vogelstein (Bunz et al., 2002)	N/A
Human: HCT116 Flp-In™ T-REx™ (male)	This study	N/A
Human: HCT116 Flp-In™ T-REx™ GFP-H2B (male)	This study	N/A

**Experimental Models: Organisms/Strains**		

N/A		

**Oligonucleotides**		

sgRNA targeting *TP53*: 5′ AAT GTT TCC TGA CTC AGA GG 3′	Horizon Discovery	N/A
sgRNAs targeting *MAPK14*: 5′ GAC AGG TTC TGG TAA CGC TC 3′; 5′ CCA TAG GCG CCA GAG CCC AC 3′	Horizon Discovery	N/A
siRNA ON-TARGETplus SMARTpool targeting HIF-1α: 5′ GAA CAA AUA CAU GGG AUU A 3′; 5′ AGA AUG AAG UGU ACC CUA A 3′; 5′ GAU GGA AGC ACU AGA CAA A 3′; 5′ CAA GUA GCC UCU UUG ACAA 3′	Dharmacon/ Horizon Discovery	Cat# L-004018-00-0005
siRNA targeting Tao1: 5′ GUA AUA UGG UCC UUU CUA A 3′	(Westhorpe et al., 2010)	N/A
Nested-PCR: *TP53* forward primers: F1 - 5′ CAG GAA GGG AGT TGG GAA TAG 3′; F2 – 5′ GAA GTG CAT GGC TGG TGAG GG 3′	This study	N/A
Nested-PCR: *TP53* reverse primers: R1 – 5′ GGA CCT GGG TCT TCA GTG AAC 3′; R2 – 5′ GAG CAG TCA GAG GAC CAG GTC 3′	This study	N/A
RT-PCR p38α-*Xho*I forward primer: CAC CTC GAG TCT CAG GAG AGG CCC ACG TTC	This study	N/A
RT-PCR p38α-*Not*I reverse primer: CAC GCG GCC GCT CAG GAC TCC ATC TCT TCT TG	This study	N/A

**Recombinant DNA**		
cDNA: MAPK14, transcript variant 2 (p38 alpha)	This study	Accession# NM_139012
pBluescript II SK- vector	Agilent genomics	Cat# 212206
pBluescript/GFP/P53-800	This study	N/A
pcDNA5/FRT/TO	Invitrogen	Cat# V652020
pcDNA5/FRT/TO/GFP-H2B	This study	
pcDNA5/FRT/TO-p38α	This study	N/A
pD1301-AD:153663 TP53_48277	Horizon Discovery	N/A
p38a sgRNA plasmid (clone 1): pD1301-AD:155747 MAPK14_25032	Horizon Discovery	N/A
p38a sgRNA plasmid (clone 2): pD1301-AD:155748 MAPK14_25033	Horizon Discovery	N/A
pOG44 Flp-Recombinase	Invitrogen	Cat# V600520

**Software and Algorithms**		

AneuFinder	(Bakker et al., 2016)	https://www.rdocumentation.org/packages/AneuFinder/versions/1.0.3
Bowtie2	(Langmead and Salzberg, 2012)	http://bowtie-bio.sourceforge.net/bowtie2/index.shtml; RRID: SCR_016368
Bravo Automated Liquid Handling Platform	Agilent Technologies	N/A
CellASIC^®^ ONIX	Merck Millipore	CAX2-S0000
ChemiDoc Touch Imaging System	BioRad	1708370
FastQ screen	Babraham Institute	http://www.internationalgenome.org/category/fastq/; RRID: SCR_000141
GelCount	Oxford Optronix	N/A
Illumina NextSeq 450 System	Illumina	RRID: SCR_014983
Illustrator^®^ CC 2018	Adobe Systems	https://www.adobe.com/uk/products/illustrator.html; RRID: SCR_010279
IncucyteZOOM^®^	Essen Bioscience	GUI = 2016A
MetaMorph^®^ Microscopy Automation & Image Analysis Software	Molecular Devices	https://www.moleculardevices.com/products/cellular-imaging-systems/acquisition-and-analysis-software/metamorph-microscopy; RRID: SCR_002368
Prism 7	GraphPad	https://www.graphpad.com/; RRID: SCR_002798
Seahorse Wave	Agilent Technologies	RRID: SCR_014526
VisionWorks^®^ LS	UVP	N/A

**Other**		

6-well plates	Corning	Cat# 353046
24-well plates	Corning	Cat# 353047
96-well black μclear^®^ plates	Greiner Bio-One	Cat# 655087
96-well clear plates	Corning	Cat# 353072
DharmaFECT 1	Dharmacon/ Horizon Discovery	Cat# T-2001-03
Dulbecco’s Modified Eagle Medium (DMEM)	Life Technologies	Cat# 41966052
EZ-Chemiluminescence Detection Kit for HRP	Geneflow Limited	Cat# KI-0172
Fetal Bovine Serum Heat Inactivated	Life Technologies	Cat# F9665
FluoroBrite DMEM media	Life Technologies	Cat# A1896701
Immobilon-P PVDF Membrane	Merck Millipore	Cat# IPVH00010
IncuCyte® Caspase 3/7 Green Apoptosis Reagent	Essen BioScience	Cat# 4440
Lipofectamine Plus	Invitrogen	Cat# 18324012
Lipofectamine 2000	Invitrogen	Cat# 11668019
Luminata Forte Western HRP Substrate	Merck Millipore	Cat# WBLUF0100
Opti-MEM	Life Technologies	Cat# 11058021
Quick Start Bradford 1x Dye Reagent	Bio-Rad Laboratories	Cat# 5000205
Seahorse XF Base Medium	Agilent Technologies	Cat# 102353-100
Seahorse XFe96 Fluxpak Mini	Agilent Technologies	Cat# 102601-100

### Contact for Reagent and Resource Sharing

Further information and requests for resources and reagents should be directed to and will be fulfilled by the Lead Contact, Stephen S. Taylor (stephen.taylor@manchester.ac.uk).

### Experimental Model and Subject Details

#### Human Cell Lines

The human, male colon carcinoma cell line HCT116 was obtained from the American Type Culture Collection (ATCC). HCT116 Flp-In™ T-REx™ derivatives were generated using the Flp-In™ T-REx™ System (Invitrogen) according to manufacturer instructions. HCT116 *TP53*^*−/−*^ cells generated by rAAV-mediated homologous recombination were provided by Bert Vogelstein (Bunz et al., 2002). To create the HCT116 Flp-In™ T-REx™ GFP-H2B cell line, an open reading frame encoding histone H2B was generated by RT-PCR amplification (Invitrogen) of mRNA prepared from HCT116 cells and cloned as a GFP-tagged fusion into a pcDNA5/FRT/TO-based expression vector (Invitrogen). This plasmid was transfected into the HCT116 Flp-In™ T-REx™ cell line using Lipofectamine Plus (Invitrogen), following manufacturer’s instructions. Cell lines were cultured in DMEM plus 10% fetal calf serum (Life Technologies), 100 U/ml penicillin, 100 U/ml streptomycin and 2 mM glutamine (all from Sigma), then maintained at 37°C in a humidified 5% CO_2_ atmosphere. All cell lines were authenticated by the Molecular Biology Core Facility at the CRUK Manchester Institute using Promega Powerplex 21 System and periodically tested for mycoplasma.

### Method Details

#### Materials and plasmids

AZ3146 (Hewitt et al., 2010), SB203580 (Tocris Bioscience), DNA-PK inhibitor II (Calbiochem), GSK923295 (Bennett et al., 2015), Nutlin-3 and nocodazole were dissolved in DMSO, stored at −20°C - except for Nutlin-3 that was stored at −80°C - and used at final concentrations of 2 μM, 10 μM, 10 μM, 100 nM, 10 μM and 0.66 ng/ml respectively unless indicated otherwise. Hygromycin B (Sigma) and hydrogen peroxide (Sigma) were stored at 4°C and used at the final concentrations of 400 μg/ml and 500 μM, respectively.

#### CRISPR/Cas9-mediated mutagenesis

For CRISPR-Cas9-mediated mutagenesis, 5x10^4^ cells were seeded per well in a 24-well plate (Corning) and maintained at 37°C in a humidified 5% CO_2_ atmosphere overnight. Transfections using Lipofectamine 2000 was performed according to the manufacturer’s instructions to transfect a pD1301-based plasmid (Horizon Discovery), which expresses Cas9, GFP and a small guide-RNA (sgRNA) targeting the gene of interest. After incubating at 37°C in a humidified 5% CO_2_ atmosphere for 48 hr, transfected cells were sorted by flow cytometry using a BD Influx cell sorter and GFP-positive cells seeded 1 cell per well in 96-well plates (Corning) to generate clonal cell lines which were then screened by immunoblotting to identify desired cell lines.

#### GFP tagging using CRISPR/Cas9

A biosensor line expressing GFP-tagged p53 was generated by co-transfecting HCT116 Flp-In T-REx™ cells with the pD1301-AD:153663 plasmid (Horizon Discovery) containing a sgRNA targeting *TP53* and a pBluescript-based plasmid containing an open reading frame encoding GFP flanked by regions of *TP53* generated by PCR amplification of genomic DNA isolated from HCT116 cells using Purelink Genomic DNA Mini kit (Invitrogen). To enrich for targeted clones, 10 days after transfection, cells were exposed to Nutlin-3 for 24 hr to stabilize GFP-p53, green fluorescent cells isolated via sorting by flow cytometry, expanded into clones then screened by immunoblotting to identify desired cell lines.

#### Targeted integration of p38α into HCT116 Flp-In cells

To reconstitute p38α function in *MAPK14*^*−/−*^, a p38α cDNA (Accession#: NM_139012) was amplified by RT-PCR using Superscript III One Step RT-PCR Platinum Taq HiFi (Invitrogen) with RNA template extracted from HCT116 cells using RNeasy Plus Mini kit (QIAGEN). This PCR product was cloned into pcDNA5/FRT/TO (Invitrogen) and transformed into XL1-Blue competent cells. Plasmid DNA was extracted using QIAprep Spin Miniprep Kit (QIAGEN) and co-transfected with pOG44 into GFP-p53 *MAPK14*^*−/−*^ HCT116 Flp-In T-REx™ cells. Following selection in 400 μg/ml hygromycin B (Sigma), colonies were pooled and expanded to create an isogenic polyclonal cell line. Expression of p38α by the addition of tetracycline hydrochloride (1 μg/ml) was confirmed by immunoblotting.

#### RNA interference

For RNAi-mediated inhibition, cells were plated in flat bottom, low evaporation 24-well plates (Corning) then transfected with a final concentration of 66 nM of the desired siRNA using DharmaFECT 1 transfection reagent (Dharmacon) in Opti-MEM® media (Life-Technologies). Knock-down was confirmed by immunoblotting.

#### Cell cycle analysis

For DNA content analysis, cells were seeded in 6-well plates (Corning) then treated with small molecule inhibitors (AZ3146, 2 μM; SB203580, 10 μM; DNA PKi, 10 μM) for 24 hr, fixed in ethanol overnight, treated with RNase A (50 μg/ml), stained with propidium iodide (40 μg/ml) then analyzed on CyAn (DakoCytomation).

#### Immunoblotting

Proteins were extracted by boiling cell pellets in sample buffer (0.35 M Tris pH 6.8, 0.1 g/ml sodium dodecyl sulfate, 93 mg/ml dithiothreitol, 30% glycerol, 50 μg/ml bromophenol blue), resolved by SDS-PAGE, then electroblotted onto Immobilon-P membranes. Following blocking in 5% dried skimmed milk dissolved in TBST (50 mM Tris pH 7.6, 150 mM NaCl, 0.1% Tween-20), membranes were incubated with primary antibodies overnight at 4°C. Note, the following monoclonal and polyclonal antibodies were used: Anti-mouse p53 (DO-1), anti-mouse p21 (F-5), anti-mouse Chk1 (G-4) (all from Santa Cruz Biotechnology); anti-mouse phospho-p38α (T180/Y182) (R&D Systems), anti-mouse Hif1α (Clone 54) (BD Transduction Laboratories), anti-mouse p38α, anti-rabbit phospho-MAPKAP-K2 (Thr334), anti-rabbit phospho-p53 (Ser 46), anti-rabbit p38 MAPK, anti-rabbit phospho-HSP27 (Ser82), anti-rabbit phospho-MKK3/6 (Ser189/207), anti-rabbit phospho-p44/42 MAPK (ERK1/2), anti-rabbit phospho-SAPK/JNK (T183/Y185), anti-rabbit phospho-Chk1 (Ser 345) (all Cell Signaling Technology); anti- rabbit ATM [Y170], anti-mouse phospho-ATM (S1981), anti-rabbit DNA-PKcs, anti-rabbit phospho-DNA-PKcs (S2056) (All from Abcam); anti-mouse phospho-KAP1 (S824) (Bethyl Laboratories), anti-rabbit phospho-gamma H2AX (S139) (Novus Bio), anti-sheep Bub3 (A. Holland and S.S.-T., unpublished data), anti-sheep Tao1 (Westhorpe et al., 2010). Membranes were then washed three times in TBST and incubated for at least 1 hr with appropriate horseradish-peroxidase-conjugated secondary antibodies (Invitrogen). After washing in TBST, bound secondary antibodies were detected using either EZ-ECL Chemiluminescence Reagent (Geneflow) or Luminata™ Forte Western HRP Substrate (Merck Millipore) and a Biospectrum 500 imaging system (UVP).

#### Cell fate profiling

To measure proliferation, apoptosis induction, and to perform cell fate profiling, 1 × 10^5^ cells were seeded per well in μclear® 96 well plates (Greiner Bio-One) and IncuCyte Kinetic Caspase-3/7 Apoptosis Assay Reagent (Essen BioScience) added. Shortly after adding inhibitors, cells were then imaged using an IncuCyte® ZOOM (Essen BioScience) equipped with a 20x objective and maintained at 37°C in a humidified 5% CO_2_ atmosphere. Phase contrast and fluorescence images (3-4 images per well) were collected every 10-30 min and IncuCyte® ZOOM software, used in real-time, measured confluence and fluorescence as a proxy for proliferation and apoptosis, respectively. Apoptosis was quantitated by measuring green fluorescence object count in 3-4 images per well, sampling at least 800 cells. Image sequences were then exported in MPEG-4 format and analyzed manually to generate cell fate profiles. Timing data were imported into Prism 7 (GraphPad) for statistical analysis and presentation. Note that 0 hr on the fate profiles represents when cells entered mitosis or when imaging started.

#### Metabolic profiling

To analyze glycolysis and oxidative phosphorylation, cells were analyzed in a Seahorse XFe96 Analyzer (Pike Winer and Wu, 2014). 1x10^3^ cells were seeded per well in Agilent Seahorse 96-well XF Cell Culture microplates and incubated at 37°C in a humidified 5% CO_2_ atmosphere overnight. Seahorse XF cartridges were hydrated with Seahorse XF Calibrant and placed in a 0% CO_2_ incubator for at least 6h. Prior to the assay, DMEM was replaced with XF Base Medium (37°C, pH 7.35 ± 0.05) supplemented with 2 mM L-glutamine only (glycolysis assay) or a mixture of 2 mM L-glutamine, 2 mM sodium pyruvate and 10 mM D-(+)-glucose (oxidative phosphorylation assay). Compound injections for different stress tests were prepared according to the manufacturer’s instructions (Seahorse XF Cell Mito Stress Test Kit and Seahorse XF Glycolysis Stress Test Kit) and added to the respective ports on the cartridge. Five measurements were made for basal metabolism and three measurements were made for each compound injection. Results were normalized for protein content in each well and assessed by Bradford assay (Bio-Rad). Note that values were derived from three independent experiments.

#### Metaphase Spreads

For chromosome counting, cells were seeded in 6-well plates (Corning) and incubated at 37°C in a humidified 5% CO_2_ atmosphere for 24 hr. Cells were treated with 0.66 μM nocodazole for 6 hr, harvested and cell pellets incubated in hypotonic buffer for 20 min at 37°C before overnight fixation in methanol:acetic acid (3:1). Samples were dropped onto glass slides and stained with Hoechst 33258 (Sigma). Images of individual metaphase spreads were taken on Zeiss Axiovert 200 with a 100x objective and chromosomes were counted manually.

#### Colony Formation Assay

For colony formation assays, 500 cells were seeded per well in 6-well plates and incubated at 37°C in a humidified 5% CO_2_ atmosphere overnight. Cells were treated with the inhibitors and washed out after 24 hr of incubation. Cells were incubated for a further 13 days to allow colony development and finally fixed in 1% formaldehyde and stained with 0.05% (w/v) crystal violet solution. Colonies were counted using a GelCount (Oxford Optronix) and imaged using a ChemiDoc™ Touch Imaging System (BioRad).

#### Single-cell whole-genome sequencing

Single G1 nuclei were isolated, sorted, and sequenced as described (Bakker et al., 2016, van den Bos et al., 2016) Briefly, cells were incubated in a cytoplasmic lysis buffer and stained with propidium iodide (10 μg/mL) and Hoechst 33258 (10 μg/mL). Single G1 nuclei were sorted in 96-well plates and stored in freezing medium at −80°C. Illumina-based library preparation was performed on a Bravo Automated Liquid Handling Platform (Agilent Technologies). Samples were sequenced on an Illumina NextSeq 450 at ERIBA (Illumina). Unprocessed sequencing reads were demultiplexed using library-specific barcodes and converted into fastq format using standard Illumina software (bcl2fastq version 1.8.4). Demultiplexed reads were aligned to human reference genome GRCh38 using Bowtie2 (version 2.2.4) (Langmead and Salzberg, 2012). Duplicate reads were marked and removed using BamUtil (version 1.0.3.). Aligned sequencing reads were analyzed and curated using AneuFinder (version 1.4.0, Bakker et al., 2016) using 1Mb bins.

#### M-FISH

To prepare chromosome spreads, cells were seeded in 6-well plates (Corning) and incubated for 24 hrs, treated with 150 ng/ml nocodazole for 4-8 hrs, then the entire population harvested by trypsinisation. Cell pellets were incubated for 25 mins at 37°C in 0.8% sodium citrate hypotonic buffer followed by overnight fixation in freshly prepared methanol:acetic acid (3:1). Samples in fixative were dropped onto glass slides and air-dried overnight at room temperature and stained using Hoechst 33258 (Sigma). Images of individual metaphase spreads were taken on Zeiss Axiovert 200 with a 100x objective and chromosomes manually counted. Slides were then experimenter-blinded and hybridized with the M-FISH probe kit 24XCyte (Zeiss MetaSystems) following the manufacturer instructions, then analyzed using an Olympus BX60 microscope for epifluorescence equipped with a Sensys CCD camera (Photometrics, USA). Images were collected and analyzed using the Genus Cytovision software (Leica). A minimum of 25 metaphases were karyotyped for each cell line/condition.

#### Time-lapse Microscopy

HCT116 Flp-In T-REx™ GFP-H2B cells were seeded in μclear® 96 well plates (Greiner Bio-One) at 1x10^4^ cells per well in FluoroBrite DMEM media (Life Technologies) plus 1 μg/ml tetracycline hydrochloride and incubated at 37°C in a humidified 5% CO_2_ atmosphere for 24 hrs. GSK923295 and AZ3146 inhibitors were added at 100 nM and 0.5 μM respectively, and time-lapse microscopy was performed on a manual microscope (Axiovert 200; Carl Zeiss, Inc.) equipped with an automated stage (PZ-2000; Applied Scientific Instrumentation) and an environmental control chamber (Solent Scientific), which maintained the cells at 37°C in a humidified stream of 5% CO2. Imaging was performed using a 40x Plan NEOFLUAR objective. Shutters, filter wheels, and point visiting were driven by MetaMorph software (MDS Analytical Technologies). Images were taken using an Evolve delta camera (Photometrics).

### Quantification and Statistical Analysis

Prism 7 (GraphPad) was used for statistical analysis, where ^∗^p < 0.05, ^∗∗^p < 0.01, ^∗∗∗^p < 0.001, ^∗∗∗∗^p < 0.0001, ns: p > 0.05. Details of statistical analyses are described in the Figure legends. Values on apoptosis and proliferation line graphs show the mean and SD or SEM from three technical replicates. Lines on scatterplots show mean and interquartile ranges. Box-and-whisker plots show the median, interquartile ranges, and the full range. To determine EACR and OCR, five measurements were made for basal metabolism and three measurements were made for each compound injection (technical replicates). Results were normalized for protein content in each well and assessed by Bradford assay (Bio-Rad). The values shown in Figure 5A show the mean and SEM from three independent biological replicates and analyzed using a two-way Anova and Tukey multiple comparisons.

### Data and Software Availability

The accession number for the raw unaligned single-cell sequencing reads reported in this paper is European Nucleotide Archive (ENA): PRJEB27319.

## Supplemental Information

Document S1. Figures S1–S7 and Table S1

Document S2. Article plus Supplemental Information
