# Supplementary material for: The p38α Stress Kinase Suppresses Aneuploidy Tolerance by Inhibiting Hif-1α
Source: Cell Rep. 2018 Oct 16;25(3):749–760.e6. doi: 10.1016/j.celrep.2018.09.060 (PMC6205844; doi:10.1016/j.celrep.2018.09.060)
Supplement: Document S1. Figures S1–S7 and Table S1 [file mmc1.pdf]

Cell Reports, Volume *25*

Supplemental Information

The p38α Stress Kinase Suppresses Aneuploidy Tolerance by Inhibiting Hif-1α

Susana Simões-Sousa, Samantha Littler, Sarah L. Thompson, Paul Minshall, Helen Whalley, Bjorn Bakker, Klaudyna Belkot, Daniela Moralli, Daniel Bronder, Anthony Tighe, Diana C.J. Spierings, Nourdine Bah, Joshua Graham, Louisa Nelson, Catherine M. Green, Floris Foijer, Paul A. Townsend, and Stephen S. Taylor

Supplemental Figures

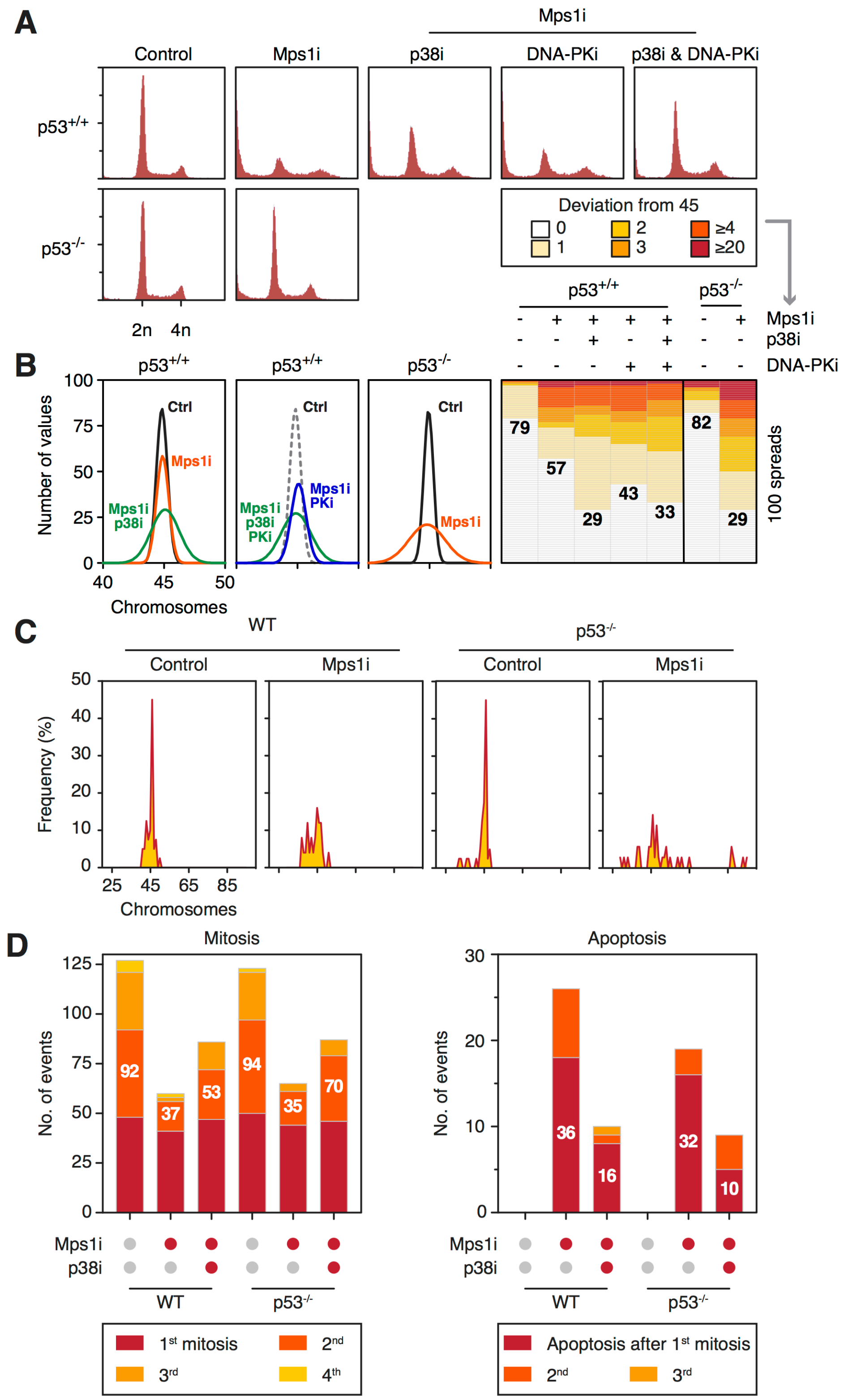

Figure S1, related to Figure 1. SB203580 suppresses apoptosis following chromosome missegregation. (**A**) DNA content and (**B**) chromosome count histograms of parental and ***TP53***$^{-/-}$ HCT116 cells, generated by AAV-mediated gene targeting, following a 48 hour exposure to AZ3146, SB203580 and NU7026, which target Mps1, p38 and DNA-PK respectively, showing the deviation from the modal count of 45. The control shown as a dotted grey line is derived from the same data as in the left panel. (**C**) Chromosome count histograms of parental and p53 CRISPR/Cas9 mutants following a 48 hour exposure to AZ3146. Note that the profile of untreated p53 mutants is similar to that of the parental cell line, and that when treated with AZ3146, near-tetraploid cells increase, consistent with loss of p53 function. (**D**) Bar graphs quantitating the number of mitoses and apoptotic events induced in parental and ***TP53***$^{-/-}$ mutant populations during a 72 hour exposure to AZ3146 and SB203580. Numbers highlight the percentage of cells undergoing a 2nd mitosis (left panel) or dying after the first mitosis (right panel).

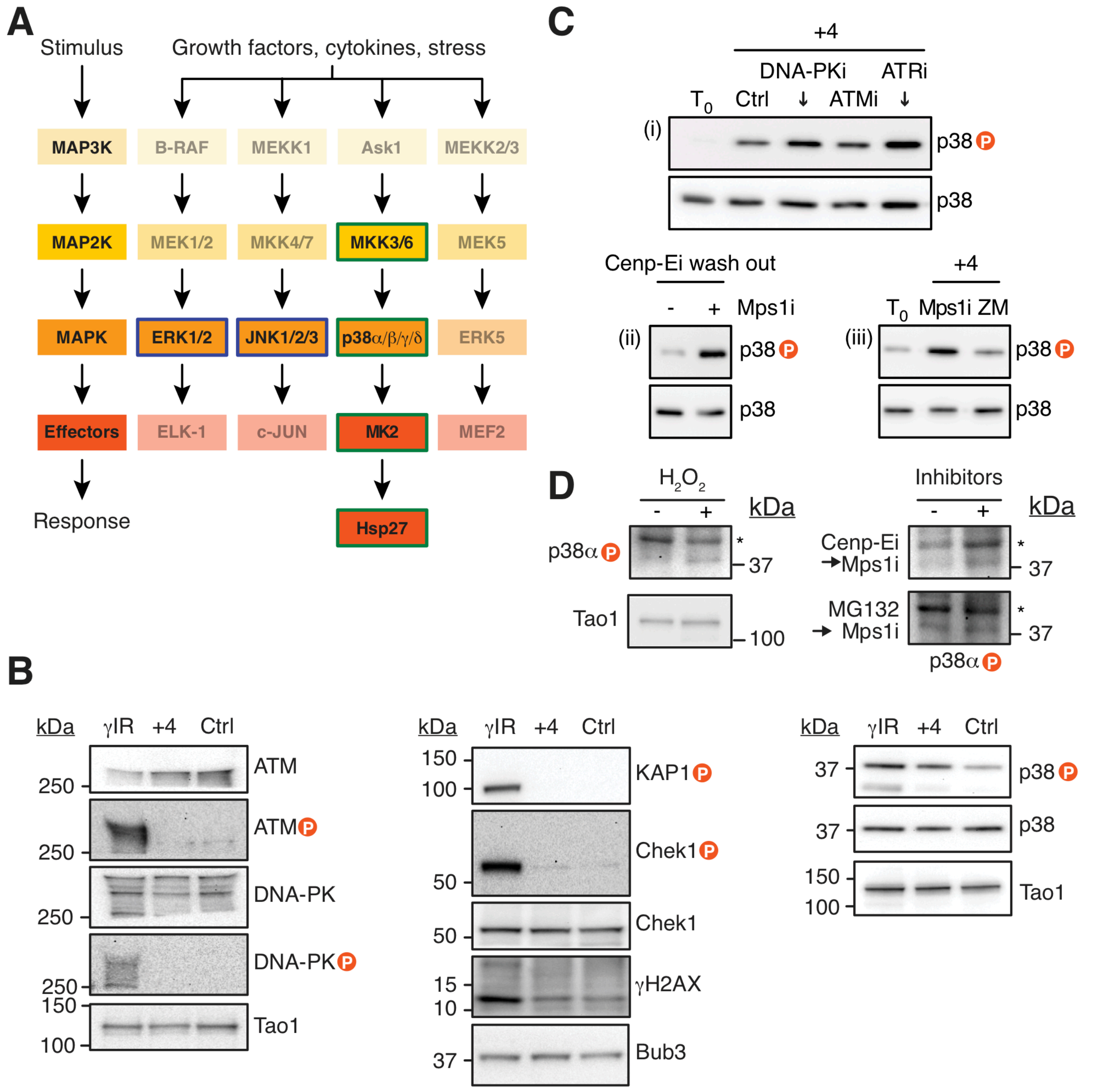

Figure S2, related to Figure 2. Aneuploidy activates the canonical p38 pathway. (**A**) Schematic representation of the ERK1/2, JNK, p38 and ERK5 pathways. In response to various stimuli, MAP3K activation leads to activation of its cognate MAP2K, in turn activating a MAPK and downstream effectors. Highlighted in green and blue are components of the canonical p38 pathway and parallel MAPKs respectively analysed in Figure 2. (**B**) Immunoblots of DNA damage response components in controls cells (Ctrl), four hours following release from sequential exposure to GSK923295 then AZ3146 (+4), and in response to 10 Gy γ-irradiation (γIR). (**C**) Phospho-p38 immunoblots of post-mitotic cells four hours following exposure to GSK923295 then AZ3146: panel (i) performed in the presence of NU7026, KU60019 and NU6027 which target DNA-PK, ATM and ATR respectively; panel (ii) washout of GSK923295 with or without AZ3146; panel (iii) mitotic exit driven by the Aurora B inhibitor ZM447439 (ZM). (**D**) Phospho-p38α immunoblots following a 30 min exposure to H_2O_2, and four hours after AZ3146-mediated override of mitotic arrest induced by either GSK923295 or the proteasome inhibitor MG132. Asterisk marks a background band.

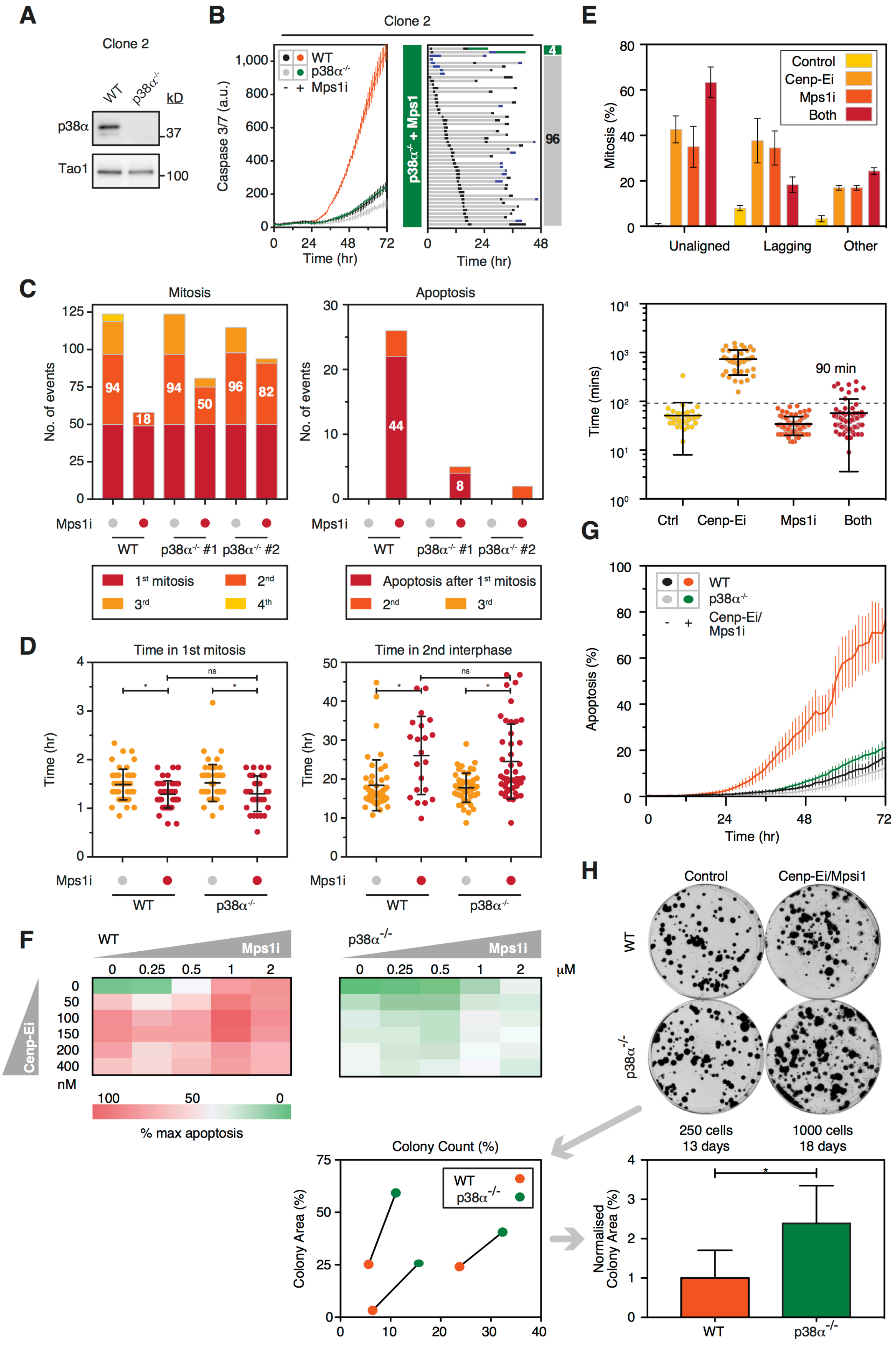

A
Clone 2
WT
p38α-/-
kD
p38α
37
Tao1
100
B
Clone 2
WT
p38α-/-
- + Mps1i
Caspase 3/7 (a.u.)
Time (hr)
p38α-/- + Mps1
4
96
E
Mitosis (%)
Control
Cenp-Ei
Mps1i
Both
Unaligned
Lagging
Other
C
Mitosis
No. of events
94
18
94
50
96
82
Apoptosis
44
8
Mps1i
WT
p38α-/- #1
p38α-/- #2
1st mitosis
2nd
3rd
4th
Apoptosis after 1st mitosis
2nd
3rd
Time (mins)
90 min
Ctrl
Cenp-Ei
Mps1i
Both
D
Time in 1st mitosis
Time in 2nd interphase
Time (hr)
ns
Mps1i
WT
p38α-/-
G
Apoptosis (%)
WT
p38α-/-
Cenp-Ei/
Mps1i
Time (hr)
H
Control
Cenp-Ei/Mpsi1
WT
p38α-/-
250 cells
13 days
1000 cells
18 days
F
WT
Mps1i
p38α-/-
Mps1i
µM
Cenp-Ei
nM
% max apoptosis
Colony Count (%)
Colony Area (%)
Normalised
Colony Area (%)

Figure S3, related to Figure 3. p38α promotes apoptosis following chromosome missegregation. (**A**) Immunoblot of CRISPR/Cas9 clone 2, generated with an independent sgRNA, showing loss of p38α. (**B**) Line graph and cell fate profile of p38α-null clone 2 showing suppression of AZ3146-induced apoptosis. Line values show mean ± SD from three technical replicates and is representative of three independent experiments. (**C**) Bar graphs quantitating the number of mitoses and apoptotic events induced in parental and p38α-null populations during a 48 hour exposure to AZ3146. Numbers highlight the percentage of cells undergoing a 2nd mitosis (left panel) or dying after the first mitosis (right panel). (**D**) Scatter plots measuring the time spent in the first mitosis (as judged by cell rounding) and the second interphase (mitosis to mitosis) for p38α-null clone 1. Note that while AZ3146 accelerates the first mitosis and delays the second interphase, loss of p38α function has no obvious additional affect. Lines represent the mean ± SD from one representative experiment. (**E**) Quantitation of mitotic errors and time spent in mitosis (nuclear-envelope breakdown to anaphase onset) following simultaneous exposure to GSK923295 and AZ3146. Bars show mean ± SEM and scatterplots shows mean ± SD from three independent experiments, except for AZ3146 alone which was analysed only twice, measuring between 29 and 121 cells per condition. Dotted line represents 90 minutes. (**F**) Heat maps, (**G**) line graph and (**H**) colony formation assay of parental and p38α-null cells simultaneously exposed to GSK923295 and AZ3146. In (F) apoptosis is measured at 72 hours, in (E, G and H) concentrations of GSK923295 and AZ3146 are 100nM and 0.5μM respectively. In (G) values show mean ± SD from two technical replicates. In (H) both colony number and area are quantitated in three independent experiments, relative to untreated controls. * $p < 0.05$.

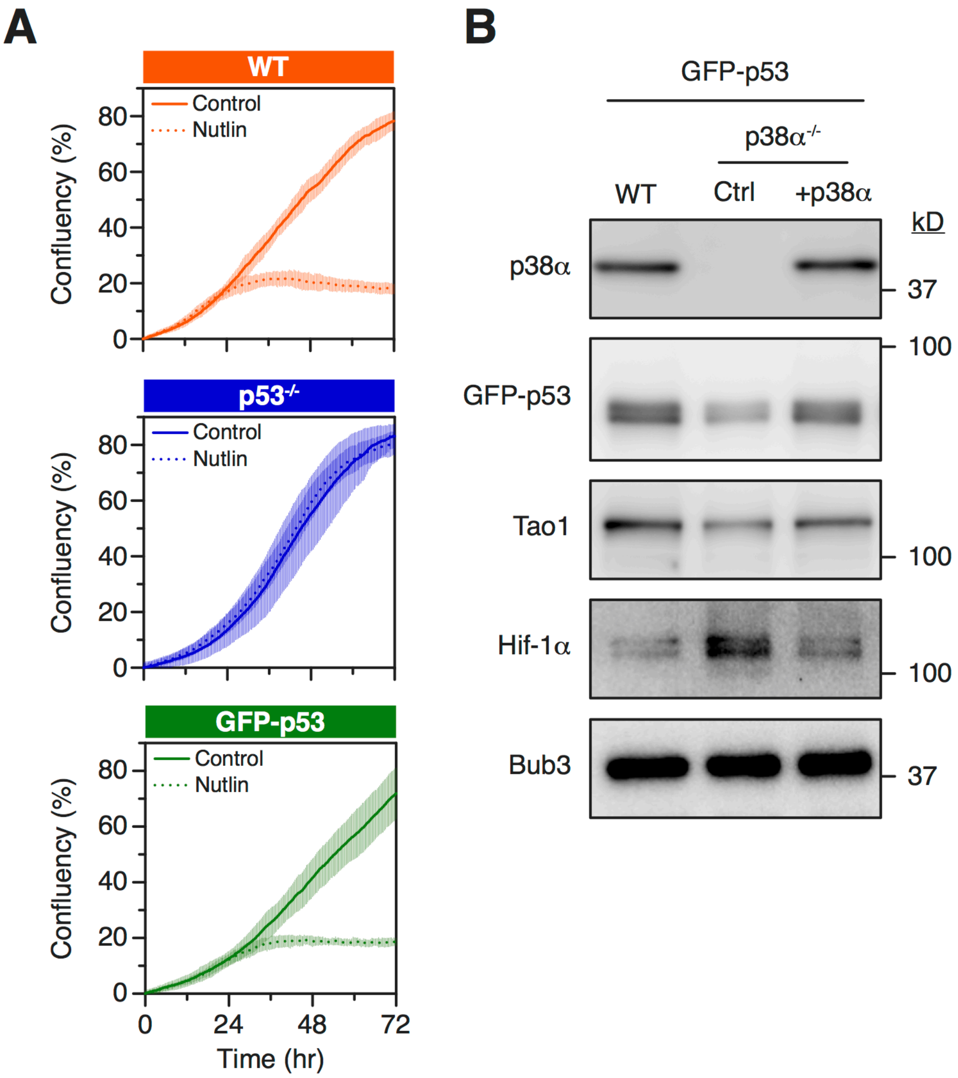

Figure S4, related to Figure 4. Characterisation of GFP-p53 cell line. (**A**) Line graphs measuring confluency as a proxy for proliferation. Values show mean ± SD derived from three technical replicates and is representative of three independent experiments. Note that while the MDM2 inhibitor Nutlin-3 suppresses proliferation of parental and GFP-p53 cells, it has no effect on p53 mutant cells, indicating that the GFP-p53 fusion is functional. Note also that Figure 4A shows p21 induction in Nutlin-3-treated GFP-p53 cells. (**B**) Immunoblot showing p38α loss following CRISPR/Cas9-mediated targeting of ***MAPK14*** in GFP-p53 cells, and subsequent restoration following Flp-mediated integration of a constitutively expressed p38α cDNA.

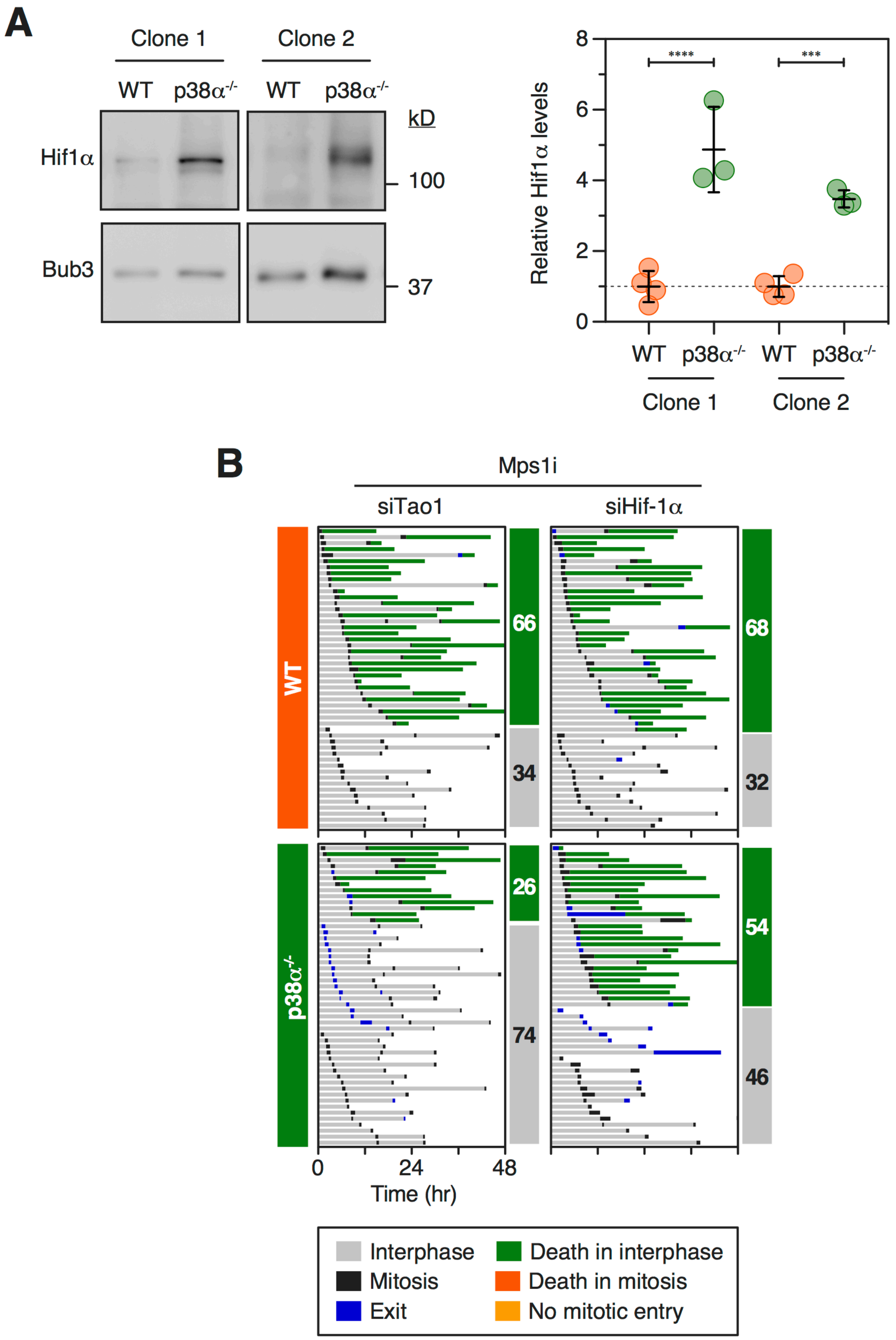

Figure S5, related to Figure 5. Suppressing Hif-1α restores post-mitotic apoptosis in p38α-null cells. (**A**) Immunoblots of Hif-1α in two independent p38α-null clones, and scatter plot quantitating Hif-1α levels, normalised to the average value obtained in parental controls. Lines represent the mean ± SD. *** $p < 0.001$, **** $p < 0.0001$. (**B**) Fate profiles showing restoration of AZ3146-mediated apoptosis in an independent p38α-null clone following siRNA-mediated repression of Hif-1α.

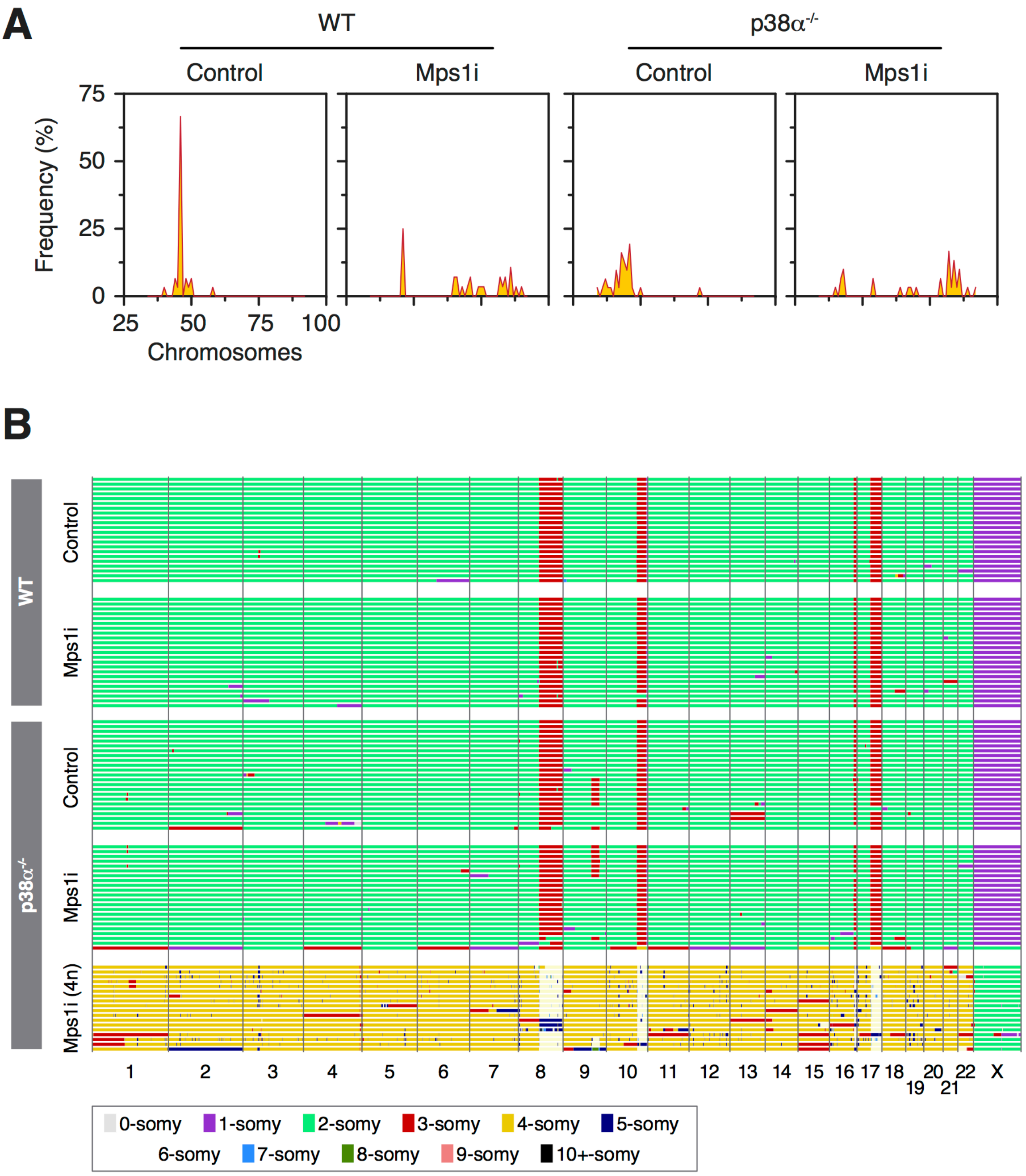

Figure S6, related to Figure 6. p38α-deficient cells accumulate whole chromosome aneuploidies. (**A**) Chromosome count histograms of parental and p38α mutant cells following a 24 hour exposure to AZ3146. Note that in contrast to the untreated p53 mutants shown in Figure S2, untreated p38α mutant cells deviate from the near diploid profile exhibited by parental cells. (**B**) Genome-wide chromosome copy number profile of parental and p38α-null cells determined by single-cell sequencing. Each row represents a single cell, with chromosomes plotted as columns and colours depicting copy number state.

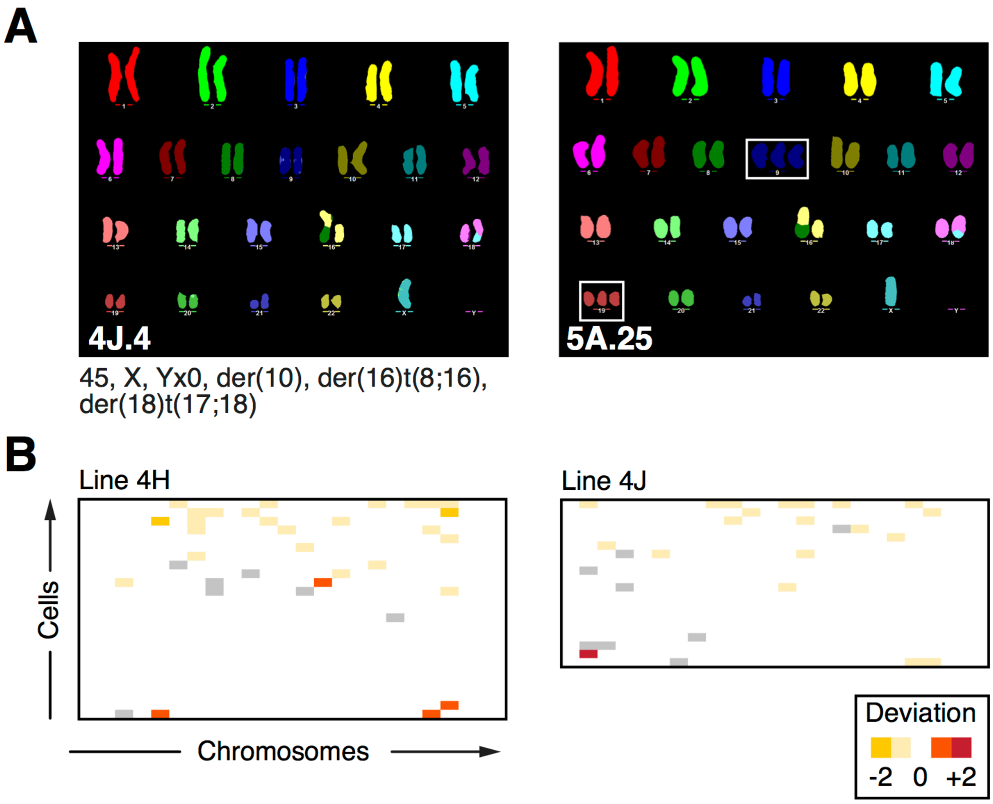

Figure S7, related to Figure 7. Pharmacological inhibition of p38 facilitates aneuploidy. (**A**) Representative M-FISH karyotypes from clones 4J and 5A. The control karyotype of 45, X, Yx0, der(10), der(16)t(8:16), der(18)t(17;18), is consistent with previously reported HCT116 karyotypes. The karyotype from clone 5A, which was generated in the continuous presence of the p38 inhibitor SB203580, is trisomic for 8 and 19 but, in contrast to the example shown in Figure 7D, disomic for chromosome 2. (**B**) Quantitation of M-FISH karyotypes from clones 4H and 4J showing little deviation from the parental line.

Supplemental Tables

	Control		Mps1i	
	WT	$p38^{-/-}$	*WT*	$p38^{-/-}$
**EACR**	95.8 ± 3.3	162.6 ± 28.1	46.2 ± 11.6	100.4 ± 38.0
**OCR**	95.5 ± 0.21	186.9 ± 14.2	66.2 ± 17.5	109.6 ± 11.5

		EACR				OCR			
		WT		$p38^{-/-}$		*WT*		$p38^{-/-}$	
		Control	Mps1i	Control	Mps1i	Control	Mps1i	Control	Mps1i
WT	Control		ns	**	ns				
	Mps1i			***	*	ns			
$p38^{-/-}$	Control				**	***	***		
	Mps1i					ns	*	***	

Table S1, related to Figure 5. p38-deficient cells show enhanced metabolic parameters. Comparison of maximal extracellular acidification rates (EACR) and oxygen consumption rates (OCR) at T=55 minutes in Figure 5A. Values in upper panel represent the mean and s.e.m. derived from three independent experiments. Lower panel shows significance derived from a two-way Anova and Tukey multiple comparisons. *** $p < 0.001$, ** $p < 0.01$, * $p < 0.05$.
